# Simple birth-death-mutation models predict some—but not all—aspects of the experimental evolution of antibiotic resistance

**DOI:** 10.1371/journal.pcbi.1014666

**Published:** 2026-08-28

**Authors:** Elin Lilja, Rosalind J. Allen, Bartlomiej Waclaw

**Affiliations:** 1 Institute of Physical Chemistry, Polish Academy of Science, Warsaw, Poland; 2 School of Physics and Astronomy, The University of Edinburgh, JCMB, Edinburgh, United Kingdom; 3 Theoretical Microbial Ecology, Institute of Microbiology, Faculty of Biological Sciences, Friedrich Schiller University Jena, Jena, Germany; 4 Cluster of Excellence Balance of the Microverse, Friedrich Schiller University Jena, Jena, Germany; University of Helsinki, FINLAND

## Abstract

Mathematical modelling of antibiotic resistance plays an important role in understanding the mechanisms of resistance emergence and spreading, testing the feasibility of new treatment protocols, and antimicrobial stewardship. However, many assumptions underlying some of the most commonly used mathematical models have not been rigorously tested experimentally. We verify whether one of these models - a birth-death-mutation process - is able to quantitatively predict the outcome of laboratory experiments. We grow bacteria in a bioreactor in conditions that closely resemble the assumptions of the model, and compare the model predictions with experimental observables such as the probability and time to resistance evolution, mutant number distribution, and the genetic composition of the evolved populations. We show that the model fails to reproduce some aspects of the experiments (failing differently for different antibiotics) but that simple modifications of the model significantly improve its predictive power. These modifications give insight into the population dynamics of resistant mutants for each antibiotic tested, and highlight the importance of quantitative modelling for accurate prediction of antibiotic resistance evolution.

## Author summary

Mathematical models are central to the study of antimicrobial resistance (AMR) because they can test mechanistic concepts, reveal processes that are difficult to observe experimentally, and predict the evolution of resistance. Surprisingly, however, these models are rarely tested against quantitative data. Here we rigorously test the predictions of one of the most widely used AMR models—the birth-death process with mutations. Using a bioreactor, we perform carefully controlled experiments in which bacteria evolve resistance to three different antibiotics. The standard, widely-used birth-death-mutation model cannot reproduce our data but augmenting it with antibiotic-specific mechanistic information can significantly increase its predictive power. Our work highlights the necessity of quantitative comparisons between AMR models and experiments. It also shows what additional factors must be considered to improve the predictions of such models, ultimately helping researchers develop new treatments that benefit from rather than being thwarted by microbial evolution.

## Introduction

Evolution of bacterial resistance to antibiotics is universally recognized as one of the most urgent problems in medicine [1–3]. Both *in vitro* laboratory models of resistance evolution [4–9] and mathematical models [10–17] are important tools for understanding the mechanisms of resistance emergence and spreading. *In vitro* models enable the study of bacterial evolution in controlled conditions which cannot be achieved *in vivo*, while mathematical models can provide insight into processes that are difficult to observe directly. These approaches are most powerful when used in combination; however, while both *in vitro* and mathematical models have been used to qualitatively explain various aspects of antimicrobial resistance, quantitative comparisons between experiments and theory are rare [18–22]. This lack of quantitative validation may lead to misinterpretation of experimental data, since, for example, it may be hard to distinguish between mechanistic models that generate qualitatively similar outcomes. Lack of experimental validation is also a barrier to the evaluation of alternative theoretical models. More extensive quantitative comparison between *in vitro* experimental data and mathematical models would greatly facilitate the development of predictive data-driven models for the emergence of bacterial antibiotic resistance.

The classic model that is often used to describe the dynamics of bacterial antibiotic resistance evolution is the birth-death-mutation model [23]. This model considers sensitive and resistant subpopulations of cells which grow and die; the growth and death rates may differ between the resistant and sensitive strains and in the presence or absence of antibiotic. Furthermore, sensitive cells mutate into resistant cells with a small probability. The main assumptions behind this model are: a well-mixed population (no spatial structure), growth/death/mutation rates independent of time, no competition between sensitive and resistant cells, all of which are violated to some extent in reality. The simple model has been extended in many ways, including non-exponential growth [24] and spatial selection gradients [25,26]. Example applications include the evolution of resistance during long-term antibiotic treatment [27], mutational pathways to resistance [28], and strain competition during combination therapy [29].

In this work we ask to what extent the birth-death-mutation mathematical model can quantitatively reproduce the evolution of antibiotic resistance in a bioreactor model of antibiotic treatment. We focus on the first step in the evolution of antibiotic resistance: the emergence and fixation of a single-point mutation that confers antibiotic resistance. We use a laboratory strain of *Escherichia coli* (the K-12 strain MG1655) and three different antibiotics: rifampicin (RIF), ciprofloxacin (CIP), and streptomycin (STR), each of which has a different mode of action. We designed our experiment to be as simple and as reproducible as possible, in order to facilitate mathematical modelling. Specifically, the population of bacteria grows exponentially, non-growing bacteria are diluted out, nutrients are unlimited, and the antibiotic concentration is at least 4x the minimum inhibitory concentration (MIC) to ensure that only resistant bacteria are able to proliferate in the presence of the antibiotic. This should eliminate mechanisms such as persistence or non-genetic resistance that could confound the results.

We show that the archetypal model of resistance evolution – the birth-death process with mutations – is able to reproduce some, but not all, aspects of the evolutionary dynamics of *de novo* resistant variants in our experiments. Moreover, different antibiotics violate different assumptions of the model. However, with the addition of simple modifications the model is able to quantitatively reproduce all of our experimental data. These modifications include: a delayed response to the antibiotic, multiple alleles having different growth rate, and phenotypic lag.

Our work confirms, reassuringly, that simple mathematical models can describe resistance evolution with quantitative accuracy. However, a quantitative description requires inclusion of the right biological mechanisms, which differ for different antibiotics. More broadly, our work shows how precise, quantitative measurements of bacterial growth combined with mathematical models can help to better understand evolutionary dynamics of bacteria and to construct better *in silico* models of AMR evolution.

## Results

### De novo evolution of resistance to rifampicin, ciprofloxacin and streptomycin in continuous cultures

**Fig 1:**
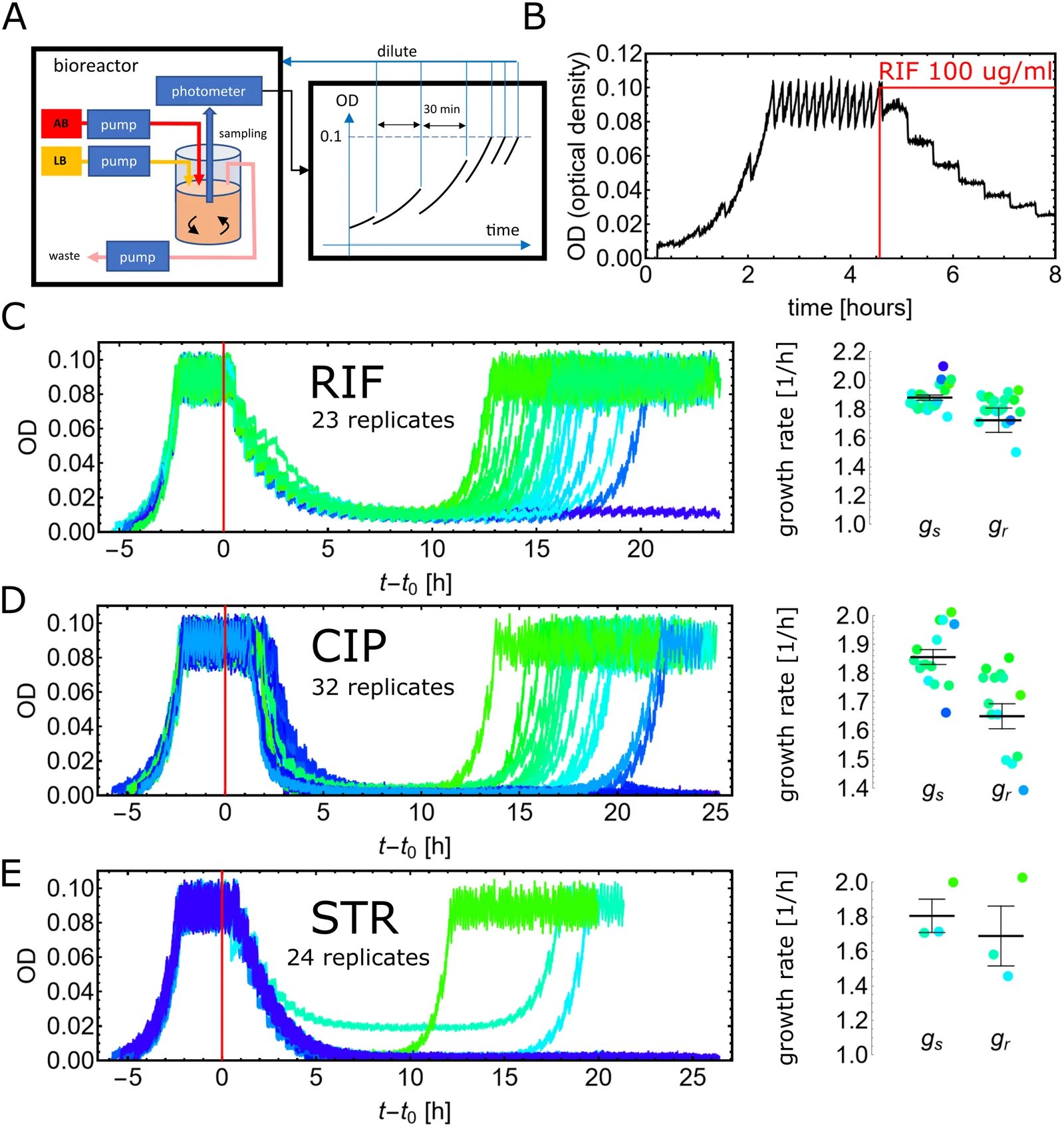
Evolution of resistance in a bioreactor. **(A)** Experimental setup. **(B)** The optical density over time in a typical experiment with rifampicin. The first part of the curve (t<2.5 h) shows exponential growth with dilution every 30 min. The second part (2.5<t<4.5 h) shows rapid dilutions that keep the OD below 0.1. The last part (t>4.5 h) shows the response to rifampicin. **(C-E)** Optical density versus time curves for all experiments, and the growth rates of sensitive and resistant populations obtained from these curves (Methods, “*Bioreactor data analysis*”), for rifampicin (C), ciprofloxacin (D) and streptomycin (E). The curves represent independent replicate experiments and have been colour-coded based on the regrowth time (green = fast regrowth, blue = slow or no regrowth). In one of the STR experiments, a problem with OD background correction caused the OD baseline to be visibly higher than zero.

We cultured *E. coli* cells in a bioreactor [30] in which bacteria, maintained in the exponential phase of growth by repeated cycles of dilution, were exposed to an antibiotic step-up (Fig 1A). Our system is similar to the turbidostat [31] and morbidostat [8] but with the differences that the optical density is allowed to fluctuate and the concentration of the antibiotic, once added, is kept constant. To achieve steady exponential growth, the culture was diluted whenever the optical density exceeded 0.1, or every 30 minutes, whichever occurred first (Fig 1B). The latter condition ensures that only actively growing bacteria are maintained in the culture; antibiotic sensitive cells, slowly-growing cells or persister cells are expected to be gradually diluted out upon antibiotic exposure. The bioreactor was inoculated with a mixture of two isogenic derivatives of the K12 *E. coli* strain MG1655: a YFP-fluorescent strain and a non-fluorescent strain, both with a deletion of the *fimA* gene to prevent biofilm formation in the culture bottles and in the cuvettes [30]. The use of a mixture of two easy-to-distinguish but identically growing strains helped to determine the clonality of the final population.

We exposed bioreactor cultures of these antibiotic-sensitive strains to three different antibiotics: streptomycin, rifampicin and ciprofloxacin, in each case at concentrations several times above their MIC (specifically, we used 100 μg/ml streptomycin, 100 μg/ml rifampicin and 100 ng/ml ciprofloxacin). These antibiotics and concentrations were selected because, in each case, *E. coli* is known to be able to achieve the corresponding level of resistance via single point mutations in the target genes of the antibiotic, i.e., in *rpsL* (streptomycin) [9,32], *rpoB* (rifampicin) [33], and *gyrA* [34] (ciprofloxacin).

Figures 1C–1E shows the optical density versus time for all our experiments, as well as the growth rates before and after antibiotic exposure. Regrowth after antibiotic exposure, indicating the emergence of resistance, occurred in 22/23, 15/32 and 3/24 of our rifampicin, ciprofloxacin and streptomycin experiments, respectively.

### A simple birth-death-mutation model cannot predict all experimental outcomes

**Fig 2:**
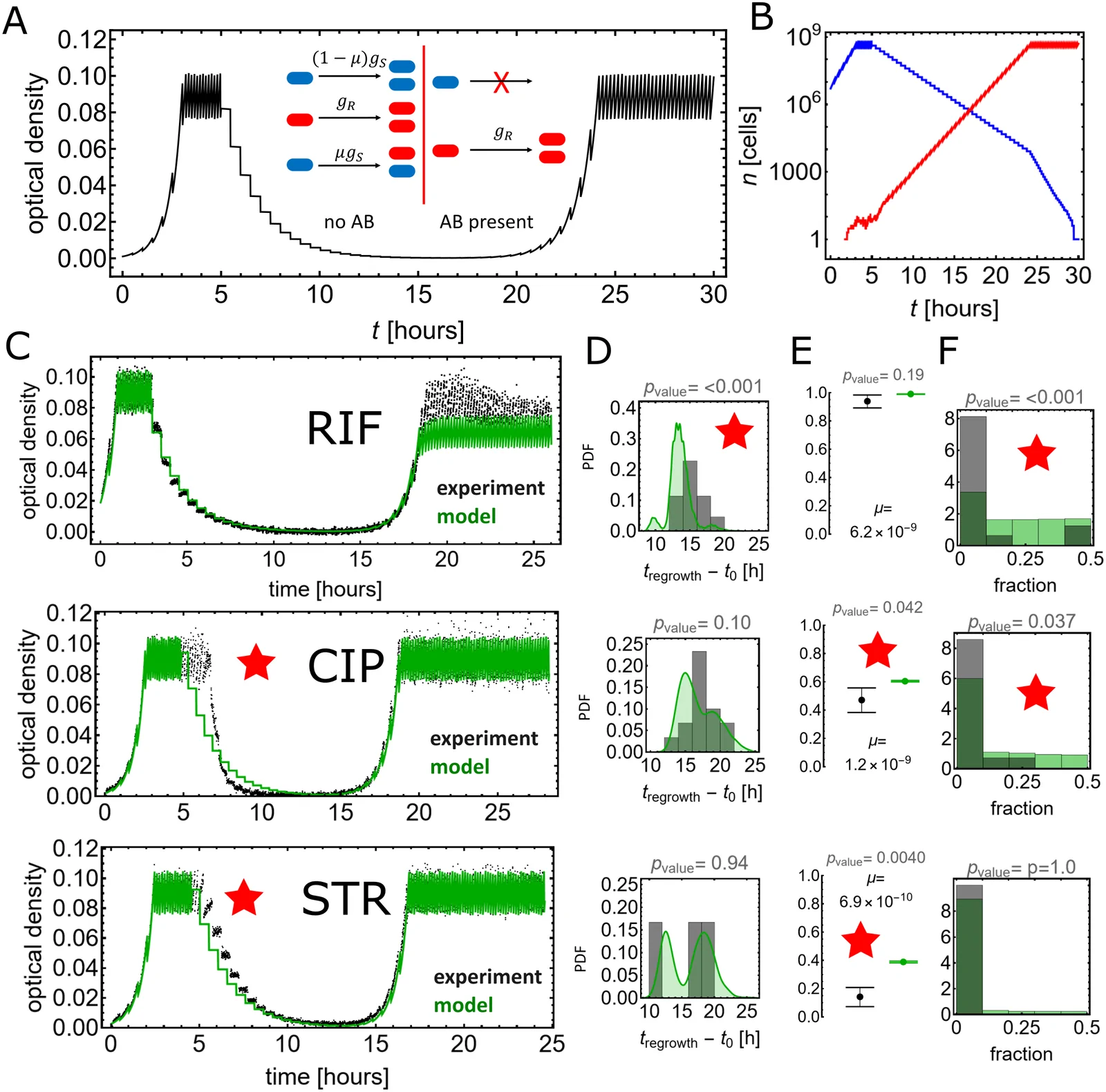
The simple model fails to predict the result of the evolutionary experiment. **(A)** Model definition and an example of optical density (OD) versus time predicted by the model. **(B)** The number of sensitive (blue) and resistant (red) cells versus time in a simulation from panel A. **(C)** Comparison of the model (green) and the experimental (black) OD versus time curves. In each case, only a single experimental and one best-fit theoretical curve has been shown. In the case of RIF we also corrected the experimental data for the contribution of the antibiotic to the optical density, since RIF and its degradation products absorb at OD600 (S1 Fig). **(D)** Probability distribution of the regrowth time for cases in which regrowth occurred. **(E)** Probability of regrowth (evolution of resistance). Error bars are Wilson score confidence intervals. **(F)** Fraction of the least abundant strain at the end of resistant regrowth. Observables that show disagreement between experiment and theory are marked with red stars.

To test whether a simple mathematical model could quantitatively explain our experimental data, we first considered the archetypal model for *de novo* evolution of antibiotic resistance: the birth-death process with mutations (Fig 2A, inset). In our version of the model, we consider the dynamics of two subpopulations of bacteria that are respectively sensitive or resistant to the antibiotic. In the absence of the antibiotic, the sensitive cells are assumed to grow with rate $g_{\text{S}}(\text{1}-\mu )$ and mutate to become resistant to the antibiotic with rate $g_{\text{S}}\mu$, whereas the resistant cells grow with rate $g_{\text{R}}$ that may be different to $g_{\text{S}}$. Hence, in this model, mutation is coupled to growth. When the antibiotic is added to the continuously growing culture, all sensitive cells are assumed to immediately cease growth while resistant cells continue to grow with the same growth rate $g_{\text{R}}$ as before.

In this model, regrowth upon antibiotic exposure can only occur due to resistant mutations that are already present at the time of antibiotic addition; *de novo* mutations do not occur after antibiotic addition because sensitive bacteria cannot replicate (and hence mutate) in the presence of the antibiotic (Fig 2B). The number of pre-existing resistant mutants depends on the population size, the number of generations before the antibiotic, and the mutation probability. Since the first two factors are the same for all three antibiotics studied, the probability of regrowth in each case can only be different if μ is different for each antibiotic.

To test whether this simple model could quantitatively match our evolutionary experiments, we ran computer simulations using experimentally-derived values of the model’s parameters: the initial number of sensitive cells, the growth rates $g_{\text{S}}$ and $g_{\text{R}}$, and the mutation probability μ. The initial number of cells was obtained from the initial optical density (Methods), assuming that there were initially no resistant cells. $g_{\text{S}}$ was determined by measuring the growth rate of the sensitive population before the addition of antibiotics and $g_{\text{R}}$ as the growth rate of the population that had re-grown after antibiotic exposure. We also factored in the periodic dilutions in our experimental system. The final parameter required was the mutation probability μ; we obtained this by performing mutation fluctuation assays [35] for each of the antibiotics used (Methods, “*Fluctuation test*”, and S2 Table). Note that, unlike in the bioreactor, bacteria are allowed to enter stationary phase before antibiotic selection in the fluctuation assays.

Figure 2A shows a typical simulated OD versus time curve; this is qualitatively in agreement with the experimental data from Fig 1C – 1E. As a first step towards a quantitative comparison between the data and the model, we superimposed simulated OD-vs-time curves on the experimental curves, selecting, for now, only such stochastic realisations of the simulation which matched the experimentally-derived regrowth times. Figure 2C shows an example of an experimental curve and a simulated curve for each antibiotic. Despite the qualitatively good match, some discrepancies are already apparent. In particular, for CIP and STR the sensitive population declines later in response to the antibiotic than predicted by the model.

To further test the model predictions, we estimated the probability that the bacterial population regrows (i.e., evolves resistance), using all our experimental and simulation data, and we also plotted the distribution (across all replicate simulations / experimental runs) of two more quantities: the regrowth time and the fraction of the least abundant strain (FLAS) at the end of resistant regrowth (Fig 2D – 2F). The regrowth time is defined as the time since the addition of the antibiotic until the optical density recovers to a value of 0.05 after the sensitive population has stopped growing and been diluted out. FLAS (a value between 0 and 0.5) is obtained by counting the number of colonies of fluorescent/non-fluorescent bacteria after plating an end-point sample on LB agar plates. A monoclonal population would have FLAS = 0 whereas a population in which two alleles have equal abundances would have FLAS = 0.5.

This quantitative comparison between the simulations and the experimental outcomes further suggests that the simple model, while qualitatively correct, cannot quantitatively predict all aspects of resistance evolution. We consider that the model disagrees with the experiments if the p-value (i.e., the probability that simulation and experimental data are statistically equivalent, for a given observable) is below 0.1. This somewhat conservative threshold p-value ensures that we only consider a model to fit the data if it performs very well, and it helps to distinguish between models that just barely reproduce the data (p-value small but ≫0.01) and models that are excellent fits ($p_{\text{value}}\approx \text{0}.\text{1}\ldots \text{1}$). For RIF, the time to regrowth (Fig 2D) is underestimated by the model and the FLAS is overestimated (Fig 2F). For CIP, the FLAS and the probability of evolving resistance are overestimated (Fig 2EF), while for STR, the probability of evolving resistance is overestimated (Fig 2E).

### Accounting for filamentation of sensitive bacteria upon CIP exposure is necessary to predict the dynamics of CIP resistance evolution

The initial decline of the bacterial population in response to antibiotic is captured correctly by the simple model only for RIF. For RIF, the apparent growth of the population ceases immediately upon exposure, as in the model (Figs 2C and S2). However, for CIP and STR the optical density continues to increase for some time after antibiotic exposure, which is not captured by the model. In the case of CIP, this effect is known to be caused by cell filamentation [36,37], (S4BD Fig): upon exposure cells continue to grow, but do not divide for some time. The continued increase in cell mass is reflected in a continued increase in the optical density even in the absence of cell division.

To model this effect, we added a time-dependent cell density-to-OD conversion factor to the model (see Methods, “*Mathematical modelling*”), which causes the OD to increase for some time post-antibiotic. This improves the model predictions for CIP (S3A and 4A Figs, column 1). In the case of STR the delayed response to the antibiotic is less significant (S3B Fig) and not caused by filamentation (S4AC Fig). However, we shall still include this factor in all subsequent STR models to ensure this observed (but very small) delay is accounted for in further extensions of the model.

### The model can predict the growth dynamics in the bioreactor in the absence of mutation

After taking into account the initial response of the sensitive population for CIP and STR, quantitative discrepancy still remains between the experimental and predicted data for some of the three measures: regrowth time, regrowth probability, and FLAS. This might suggest that either growth or mutation is incorrectly accounted for by the simple model. To test the correctness of the growth component of the model, we ran “standing variation” experiments in which we inoculated bioreactor cultures with a mixture of resistant bacteria (harvested from previous evolution experiments) and sensitive bacteria in a known proportion (about 1:1000, the exact ratio was determined by plating). A relatively high abundance of the resistant mutant was used to negate any contribution from spontaneous mutations. The model - with the delayed-response for STR and CIP included - provided predictions in very good agreement with the data for all antibiotics (S5 Fig). Hence, growth dynamics is correctly represented in the model and the quantitative discrepancies identified earlier likely arise from incorrect modelling of *de novo* mutations and the transition from genetic to phenotypic resistance.

### The generation of CIP and STR resistant clones cannot be predicted using the simple model with a mutation rate determined by a fluctuation test

**Fig 3:**
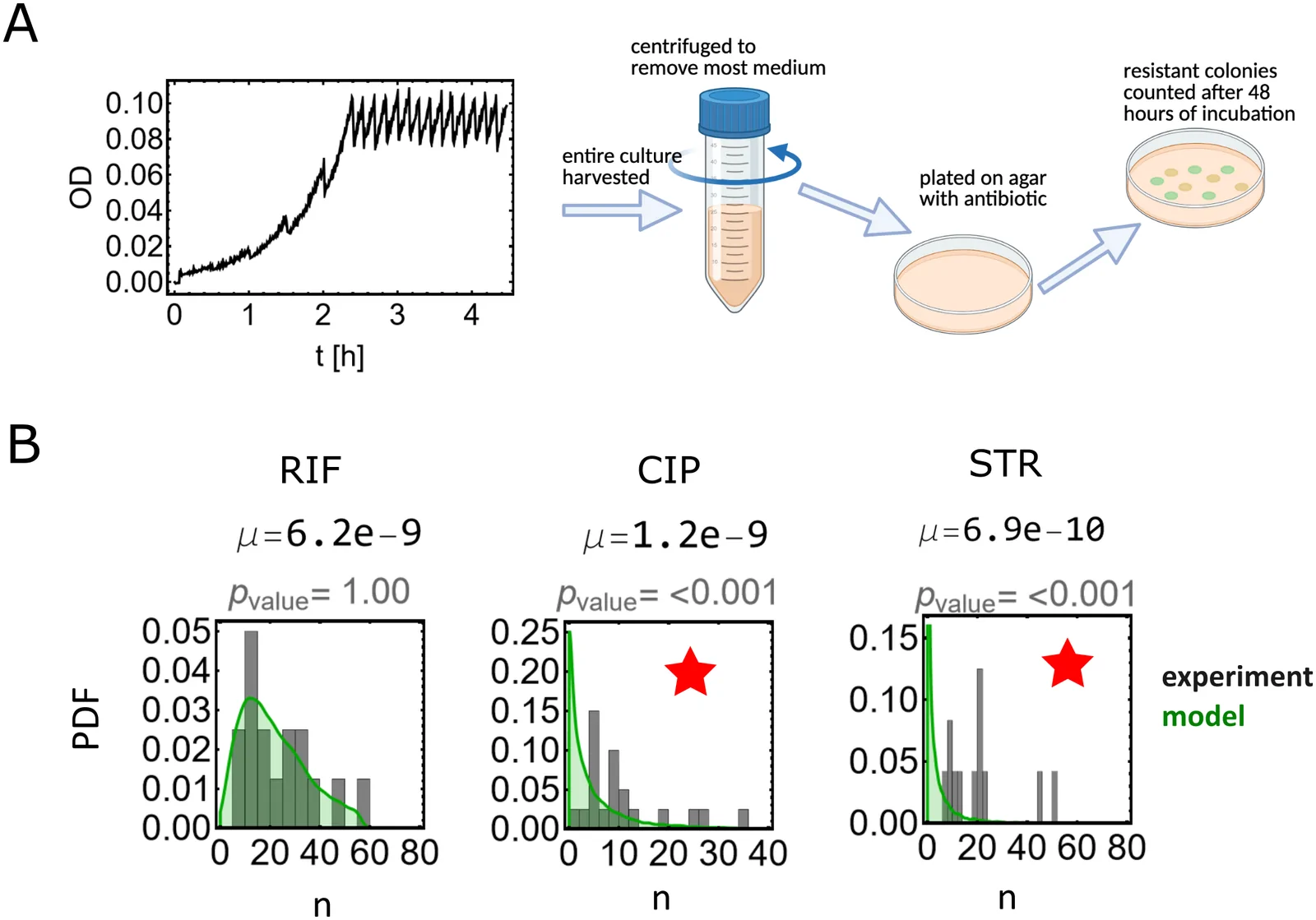
Simple model fails to predict the pre-antibiotic mutant numbers for CIP and STR. **(A)** Schematic of the experiment. Antibiotic-naive bioreactor cultures are spun down and plated on antibiotic-selective agar plates. **(B)** The distribution of the number of resistant colonies obtained experimentally (grey) and predicted by the simple model (green). Disagreement between experiment and theory is marked with a red star. Panel A has been created in BioRender (https://BioRender.com/18kxoi9).

To test the mutation component of the model, we performed further, “short” experiments to measure the accumulation of *de novo* mutations in the bioreactor. In these experiments, we stopped the bioreactor at the time when the antibiotic would normally have been added in the previous experiments, i.e., two hours after the threshold OD was reached (Fig 3A). We then plated all the cells from each culture onto an LB agar plate containing one of RIF, CIP, or STR antibiotics. The number of colonies corresponded to the number of resistant mutant cells that were present in our previous experiments at the moment when the culture was exposed to antibiotics; replicate experiments produced the distribution of mutant numbers. Simulating the same scenario with our model using the mutation rates previously measured in the fluctuation assays, we compared our experimental and simulated mutant number distributions. We found that the simple model could predict the mutant number distribution for RIF, but for CIP or STR the model underestimated the number of resistant bacteria that were present in the bioreactor (Fig 3B).

This discrepancy suggests that the mutation probability that was measured in the fluctuation assays is not an accurate estimate of the actual mutation probability in the bioreactor. This could be due to the difference in growth conditions: in the bioreactor, cells are maintained in steady exponential growth whereas in the fluctuation assay they experience exponential growth followed by stationary phase. We therefore determined the “true” mutation probability μ by fitting the model to the mutant number distributions from the bioreactor experiments (Fig 3B). For RIF, the estimate for the mutation probability obtained in this way was very similar ($\mu =\text{8}.\text{3}\times {\text{1}\text{0}}^{-\text{9}}$) to that estimated by the fluctuation assay ($\mu =\text{6}.\text{2}\times {\text{1}\text{0}}^{-\text{9}}$). However, for CIP and STR the estimated mutation probabilities from the bioreactor data ($\mu =\text{2}.\text{8}\times {\text{1}\text{0}}^{-\text{9}}$ and $\text{4}.\text{7}\times {\text{1}\text{0}}^{-\text{9}}$ respectively) were significantly higher than the values from the standard fluctuation assays ($\mu =\text{1}.\text{2}\times {\text{1}\text{0}}^{-\text{9}}$ and $\text{6}.\text{9}\times {\text{1}\text{0}}^{-\text{1}\text{0}}$, respectively).

**Fig 4:**
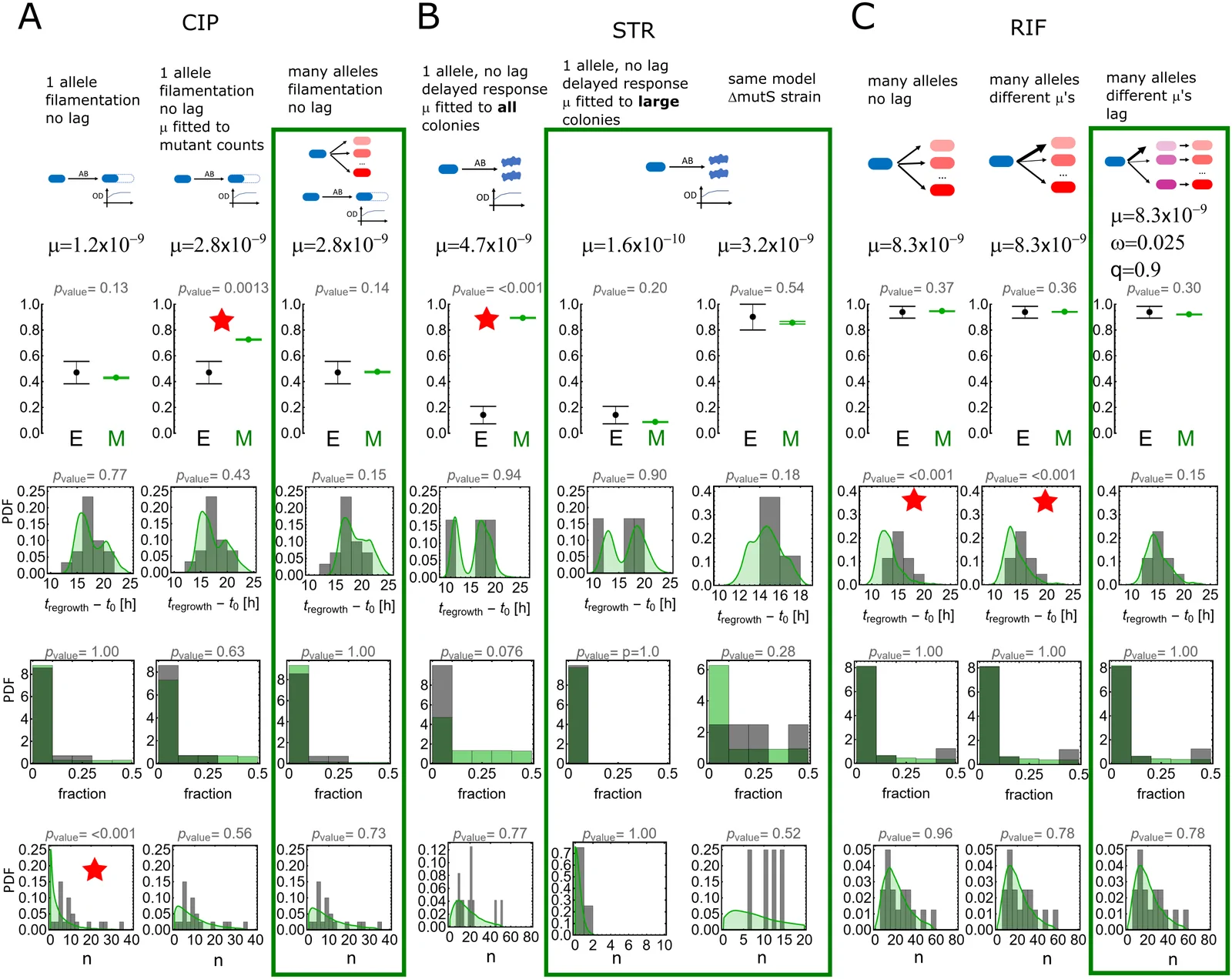
The comparison of different models to the experimental data for ciprofloxacin (A), streptomycin (B) and rifampicin (C). Rows of plots show: the probability of resistant regrowth, the time to regrowth, the fraction of the least abundant strain at the end of resistant regrowth, and the distribution of the number of resistant mutants prior to antibiotic exposure. Grey and green histograms are the experimental and simulated data, respectively. Model versions for which all experimental (“E”) and theoretical (“M”) results statistically agree (p-value >0.1) are surrounded with green rectangles. Observables that show disagreement between experiment and theory are marked with red stars.

Next, we performed simulations of the full experiment, including antibiotic addition, using the “true” mutation rate values listed above. To our surprise, this did not improve the model’s performance. For CIP (Fig 4A, column 2) the model now overestimated the probability of resistance evolution. The disagreement was even more dramatic for STR, for which the model now also generated an incorrect FLAS prediction (Fig 4B, column 1).

### Different phenotypes of resistant alleles need to be taken into account to understand the antibiotic resistance evolution experiments

Our simple model accounts for only one type of resistant mutant (for each antibiotic), but in our STR and RIF experiments we observed differently sized colonies on selective antibiotic-containing agar plates, suggesting the presence of phenotypically different resistant alleles within a single experiment. We therefore decided to include multiple resistant alleles in the model. To do this, we began by characterizing the phenotypes and genotypes of individual clones isolated in our experiments. We included clones both from the long experiments (Fig 2) and the short experiments (Fig 3).

Using whole-genome sequencing (WGS), we found that in STR-exposed clones, STR resistance was conferred either by SNPs in *rpsL* (30S ribosomal subunit protein S12, STR target), or by large deletions of 100–150 genes between positions 250 kbp and 400 kbp and no SNPs elsewhere on the MG1655 chromosome. CIP resistance was conferred by SNPs in *gyrA* (DNA gyrase subunit A) or in *gyrB* (DNA gyrase subunit A), both targeted by fluoroquinolone antibiotics. RIF resistance was conferred by SNPs in *rpoB* (beta subunit of bacterial RNA polymerase, RIF target). S1 Table lists all these mutations.

With the exception of the large-deletion strains, resistance was always conferred by single mutations in the target of the antibiotic in our experiments. The clones with the large deletion could only be isolated from “short-term” experiments, in which they formed very small colonies on STR agar plates. We concluded that these clones grew too slowly to persist in the long-term evolutionary experiments in the bioreactor. On the other hand, all STR-resistant *rpsL* clones grew at the same rate as the parent strain.

To further investigate the diversity of the resistance mutations to CIP and RIF, we measured the growth rate of all the different clones (identified by WGS) in isolation in the bioreactor in the absence and presence of the antibiotic. The growth rates of RIF and CIP mutants are summarized in S1 Table.

Since the large-deletion alleles cannot persist in the bioreactor, we concluded that the STR model of bioreactor evolution should take into account only the *rpsL* alleles. Therefore, we obtained a new estimate of the “true” mutation probability for STR by fitting our model to the colony counts from the short STR experiment, taking into account only the normal-sized colonies and ignoring the small colonies. Using this lower mutation rate ($\mu =\text{1}.\text{6}\times {\text{1}\text{0}}^{-\text{1}\text{0}}$) the model could now accurately predict the results of evolution in the bioreactor (Fig 4B, column 2).

Regrowth on STR was very rare in our long experiments, likely due to the rarity of *rpsL* mutations. This limited the size of our dataset for quantitative comparison to the model. To improve the statistics, we decided to repeat all experiments using a strain with a higher mutation rate, which we created by deleting the *mutS* [38] gene. These new experiments showed regrowth in all cultures in long experiments, and short experiments with selection on agar plates yielded both normal and small colony variants, as for the WT strain. Fitting the mutation probability to the colony counts in the short experiments, using only the normal-sized colonies produced a value of $\mu =\text{3}.\text{2}\times {\text{1}\text{0}}^{-\text{9}}$. Incorporating this mutation probability into our model including the initially delayed response yielded a single-allele model that could accurately predict all experimental outcomes for the Δ*mutS* strain (Fig 4B, column 3).

In the case of CIP and RIF, however, our growth rate measurements showed that all alleles could persist in the bioreactor, although the growth rate varied among alleles in both the presence and absence of the antibiotic. Therefore, for CIP and RIF we modified the simple model by including the dynamics of all n alleles ($n_{\text{RIF}}=\text{2}\text{1},n_{\text{CIP}}=\text{9}$), using the experimentally measured growth rates. We initially assumed that all alleles are generated with the same mutation probability $\mu /n_{\text{RIF}}$ or $\mu /n_{\text{CIP}}$. We obtained the mutation probability by fitting the model to the mutant number distribution obtained by selection on agar plates in our short experiments, taking all colonies into account. This new model with multiple alleles could now quantitatively predict all experimental outcomes for CIP (Fig 4A, column 3), suggesting that the inclusion of delayed initial response plus multiple alleles was sufficient to quantitatively explain the dynamics of evolution of resistance to CIP. However, the model still underestimated the time to regrowth for RIF (Fig 4C, column 1).

Focusing now on RIF, we next questioned our assumption that all alleles are equally likely, since it is known that mutations involving different base pair changes occur with different probabilities in *E. coli* [39,40]. Using the probabilities obtained from Refs. [39,40] in the multiple allele model improved its performance for the distribution of RIF regrowth times, but the p-value for the comparison between model and experiment was still below 0.001, suggesting significant disagreement (Fig 4C, column 2).

### The addition of phenotypic delay can explain the dynamics of RIF resistance evolution

Thus far our models assumed that a mutation immediately generates a fully resistant cell that grows with the population growth rate measured for that allele. However, it is known that some antibiotics (including the ones we use here) exhibit a phenotypic delay in the emergence of resistance [41,42]. This occurs in cases where antibiotic sensitivity is phenotypically dominant over resistance [43,44]. Immediately after the genetic mutation, a cell contains both resistant and sensitive variants of the target protein; the sensitive variants must be diluted out by growth and cell division before the cell assumes a fully resistant phenotype. Thus, there is a time delay before newly generated mutants become fully resistant. Although our experimental setup does not allow for direct measurement of this “phenotypic delay”, we hypothesized that it might account for the remaining difference between the model predictions and our RIF experimental data.

To explore this, we added a simple phenotypic delay to our model in the form of an intermediate, semi-resistant phenotype. This new phenotype grows slower than a fully resistant one (e.g., due to the sensitive protein variant still present in the cell), but faster than the sensitive phenotype. The phenotype is created from the sensitive strain with mutation probability μ but in the presence of the antibiotic its growth rate is only a fraction q of the growth rate of the fully resistant phenotype. The intermediate phenotype switches to the fully resistant phenotype with rate ω ($\gg \mu g_{\text{S}}$), with 1/ω representing the average length of phenotypic delay. This model is a crude approximation of more accurate mechanistic models of phenotypic lag [41,42].

To find the best values for ω and q, we fitted the model to all our experimental observables from the long, evolutionary bioreactor runs (Methods, “*Optimal phenotypic lag parameters*”). Adding a long but weak phenotypic delay (1/ω~40 h, q=90% of the resistant growth rate) to the multiple allele model of RIF evolution improved its fit to the experimental data (Fig 4C, column 3), such that the model could now reproduce the distribution of regrowth times with a p-value of above 0.05 (both with and without the inclusion of different mutation rates for different alleles, cf. S6 Fig). Although the CIP data could be explained (with p-values above 0.1 for all experimental observables) without a phenotypic delay, models with a short but phenotypically strong delay (1/ω=2…3 h, q=0...30%) also improved the agreement for this antibiotic (S7 Fig). We stress that the numerical values of the delay are difficult to interpret biologically because our model is not mechanistically correct; rather, it should be considered a simple, effective model of the emergence of phenotypic resistance from genetic mutations.

### Broader implications

The simple model fails in different ways for different antibiotics, each time providing an insight into what factors are important when modelling resistance. Accounting for these factors is essential for developing accurate, quantitative, and predictive models of resistance evolution.

To illustrate how these factors could affect the probability of resistance evolution more generally, we shall consider a treatment with a “generic” antibiotic, loosely based on (but not replicating the exact behaviour of) the antibiotics studied here. We shall consider each factor (phenotypic lag, multiple alleles, filamentation) in isolation, and model two scenarios: (i) exponential growth relevant for an early infection, (ii) steady state growth with population turnover. The latter could represent an established infection or an experimental model with either manual or automated serial dilutions (as in our experiments). In the simulations presented below, the parameters and antibiotic exposure duration have been chosen so that the simple model yields a 50% probability of regrowth. We also assume the same dilution factor and timing as in the experiments described here.

**Fig 5:**
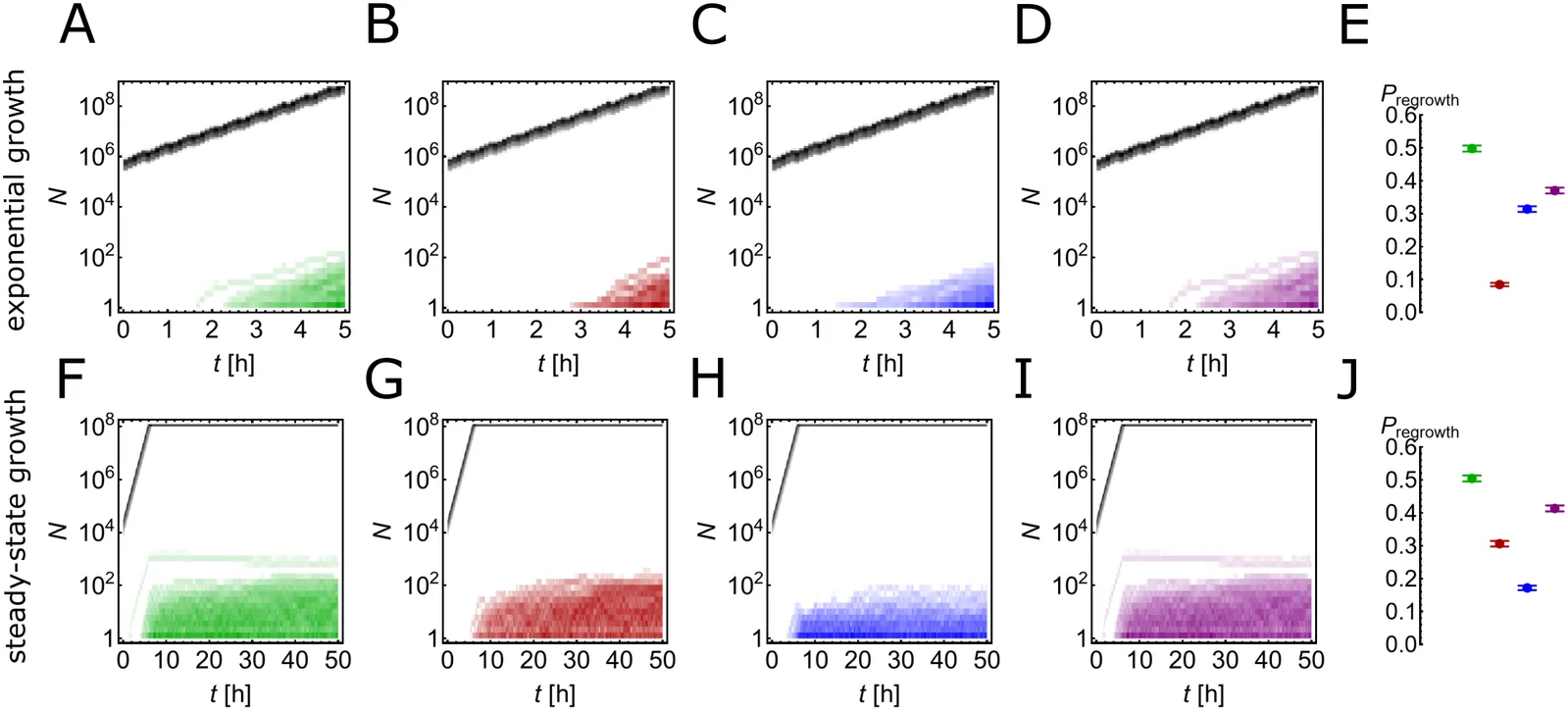
The effect of phenotypic lag, multiple alleles, and filamentation on the outcome of simulated treatment. **(A-E)** Exponential growth. **(F-J)** Steady state growth. (A, F): the simple model; (B, G): phenotypic lag; (C, H): multiple alleles model, (D, I): filamentation model. Each panel shows the sensitive population (black) and the fully resistant population (colour) as a density map obtained from 300 independent simulations. Panels (E, J) show the regrowth probability for each model, with the antibiotic added at t=5 h (exponential growth) and t=50 h (steady-state growth).

**Phenotypic lag.** We assume ω=0.04 and q=0 so that the intermediate phenotype is fully sensitive to the antibiotic, in contrast to RIF, for which q>0. In an exponentially growing population, phenotypic lag causes the number of phenotypically resistant mutants to be less than the no-lag model would predict at the same time point (Fig 5AB). This dramatically reduces the regrowth probability to 9% (Fig 5E). The difference disappears as the population approaches a steady state (we simulated up to t=50 h) with an approximately constant population size; this is because as time progresses, the fraction of resistant phenotypes also approaches a steady state (Fig 5G) determined by the mutation rate but not affected by the transition rate from genetic to phenotypic resistance, as all mutants eventually become resistant, given enough time. The post-antibiotic regrowth probability is only slightly reduced to 30% (Fig 5J).

**Multiple alleles with different growth rates.** We use the growth rates of different RIF alleles for this simulation. Exponential growth affects the proportion of different alleles; their overall number is determined mainly by the growth rate of the fittest allele. Since we still dilute the culture periodically, very unfit alleles are eliminated even in the case of exponential growth (Fig 5C), reducing the regrowth probability to 31% (Fig 5E). An even stronger selection in the steady-state simulation makes only the fittest allele survive, and the regrowth probability is 17% (Fig 5HJ).

#### Filamentation.

We assume the same delayed growth response as in the CIP model. Filamentation makes only a small difference in the case of both exponentially growing and steady-state population (Fig 5DI). The only effect is the competition between the filamenting sensitive cells, and resistant cells, reducing the fixation probability of resistant phenotypes to 37% (exponential) and 41% (steady-state) (Fig 5EJ).

This brief discussion is not intended as a comprehensive exploration of the extended models’ parameter space, but rather as a brief guide to the expected behaviour when individual new features are introduced. Moreover, these illustrative examples should be interpreted as hypotheses generated by the model, rather than general predictions applicable to all models.

## Discussion

Simple birth-death-mutation models are widely used to predict the dynamics of antibiotic resistance evolution, but it is not clear how quantitatively accurate their predictions are. In this work we performed exhaustive tests of our ability to model resistance evolution, using a quantitative dataset from a bioreactor setup that was optimized to facilitate the comparison with models, in that bacteria are kept in steady state growth and cell density and growth rate can be accurately measured, as well as the timing of resistance emergence. Our results show that although a simple birth-death-mutation model can accurately predict some aspects of the experimental evolution of antibiotic resistance, it fails to quantitatively predict all experimental outcomes. We find that the model fails for reasons that are different depending on the antibiotic used. This in turn provides insight into the mechanism of AMR evolution for each antibiotic.

The model with a single resistant allele, a mutation probability obtained from a standard mutation fluctuation test and accounting for a delayed initial response due to filamentation of the sensitive population can actually predict the outcome of resistance evolution to CIP: the probability of regrowth, and the time to regrowth as well as the fraction of the least-abundant allele. However, this model fails to predict the number of resistant mutants present before antibiotic exposure. Explicitly including different resistant alleles with experimentally measured growth rates is necessary to fully explain all the data, including the mutant number distribution.

In the case of STR, for our wild-type *E. coli* strain the probability of resistance is very low (Fig 2D), and thus we had fewer experimental datasets to compare the model to. However, we find that neither the mutation rate obtained from a standard mutation fluctuation test, nor a revised mutation rate obtained from counting colonies isolated on STR plates from growth in the bioreactor, accurately predicts the regrowth probability. This is because mutations generate an abundance of slowly-growing resistant variants conferred by large genomic deletions but relatively few fast-growing alleles in the STR target *rpsL*. Interestingly, large deletions detected by WGS always included the *hemB* gene (delta-aminolevulinic acid dehydratase), and *ΔhemB* strains are known to produce the “small-colony variant” we observed. Although the *ΔhemB* variants are known to persist in infections [45], their slow growth causes them to be washed out of the bioreactor and thus do not contribute to the evolution of resistance. In contrast, in the standard fluctuation assay, bacteria are grown in batch cultures and not being diluted out, and thus *rpsL* mutations which are STR-dependant are more likely to be selected for [32,46].

Finally, for RIF, evolution of resistance occurred in almost all long-term experiments, thanks to the large target size (many different SNPs in *rpoB* can confer resistance, see S1 Table) [33]. However, including the 21 different alleles of *rpoB* that we identified in our experiments together with their different mutation probabilities was not enough to fully explain all the experimental outcomes. Of course, our model with multiple alleles does not take into account all the possible *rpoB* mutations that can lead to RIF resistance [47,48], but considering the diversity in growth rates already included it is unlikely that including more alleles would improve the model. In particular, the model underestimated the time it takes for a resistant population to regrow in the bioreactor (Fig 4C). We therefore explored the phenomenon of phenotypic delay [42]. It has been experimentally demonstrated that some resistance mutations in *rpoB* as well as *rpsL* and *gyrA* cause phenotypic delay due to polyploidy, in which the multiple chromosomes in fast growing cells have to be diluted out before phenotypic resistance to the antibiotic can occur [41]. We modelled this in a very simplistic way by introducing an intermediate phenotype occurring upon mutation, which grows in the presence of the antibiotic at a slower rate than the resistant phenotype, and which slowly transitions to full resistance. In reality the delay will depend on cells’ history: the number of resistant/sensitive copies of the target gene, the number of resistant/sensitive target molecules, position in the cell cycle, and other factors. Nevertheless, this simple model substantially improved the agreement with the RIF data.

Our findings for RIF do not constitute a proof of phenotypic delay, nor do they explain its mechanism. Nevertheless, the model with phenotypic delay can be treated as an effective model of resistance to RIF that explains all our experimental results. Our results do not rule out phenotypic delay in the case of CIP and STR; they only show the delay is not required to model our experimental data. This could be due to the delay being very short, or not contributing to the observables we chose to look at. For example, a delay that would cause bacteria containing sensitive target molecules not to grow at all (q=0) would most likely manifest itself as a lower effective mutation probability in both the fluctuation test and the short-term bioreactor experiment. Detecting this type of delay would require a different experimental approach to that used here, possibly involving single-cell imaging [41]. Interestingly, the mutation rates for STR and CIP that we measured in the standard fluctuation assays were significantly lower than the mutation rates we obtained from the bioreactor experiments. This suggests that continuous steady state growth may contribute to the increase in the emergence of phenotypically resistant alleles. One possible explanation is that in the bioreactor, cell lineages may have more time (generations) to overcome a phenotypic delay that would otherwise prevent growth upon selection. That is, in a batch culture in the standard fluctuation assay, most mutations would likely occur at the highest cell densities when nutrients are almost depleted, allowing for few generations after the mutation to dilute out sensitive chromosomes and proteins.

Taken together, our results show that a simple birth-death-mutation model cannot predict all the features of the dynamics of resistance evolution in a bioreactor, even though our experiments were designed to be as simple as possible (i.e., with as few unmeasurable/ uncontrolled variables as possible). This is perhaps not entirely unexpected, but we think this point is worth articulating to ensure that this simple model is used with caution when applied to real data. Our work clarifies the scale of deviations that can be expected when extensions beyond the birth–death–mutation model are ignored.

We stress that the alternative models we propose have not been selected in any formal way. In particular, we do not consider alternative models that may adequately reproduce the data. For example, phenotypic lag could be modelled in a more realistic way by considering different biochemical mechanisms [42]. Cell filamentation could be modelled explicitly by considering a population of cells of different sizes depending on the internal state of the cell. Different alleles could have different phenotypic lags, cell sizes, etc. Since it was not our intention to provide the best possible model but rather to propose a model that fits the data better than the simple birth-death-mutation model, we did not consider these alternatives.

It should be noted that whether the simple model can quantitatively predict experimental outcomes depends on which outcome is looked at, and which outcomes can be accurately predicted, and the necessary modifications to the model, depend on which antibiotic is used. Indeed, accurate predictions for all models of antibiotic resistance may depend on prior knowledge of the biological processes governing a particular antibiotic–pathogen system, which can differ substantially between contexts. Thus, we conclude that, when modelling antibiotic treatment, the initial response of the population, the fitness of different resistant alleles, and phenotypic delay should be considered as important factors that may significantly affect the model prediction. Not accounting for these factors can lead to models that appear correct at a qualitative level, but fail to quantitatively predict bacterial growth and dynamics, which compromises their usefulness for modelling personalized antibiotic therapy [49].

## Materials and methods

### Bioreactor setup

We use a bioreactor system described before in Ref. [30] that maintains approximately constant turbidity of the culture by repeatedly diluting it with fresh medium using a negative feedback loop. This keeps the bacteria in the exponential growth phase in the absence of antibiotics. Our system is close to a turbidostat, however the turbidity is allowed to fluctuate in a range between OD = 0.075 and OD = 0.1 to enable fitting an exponential function to segments of OD vs time curve; this gives an accurate estimate of the growth rate.

The system consists of four replicate cultures with an approximate working volume of 25 mL each. Each bottle has two inlets: one for the LB medium, the other for the LB + antibiotic, an outlet for removing excess liquid, a sampling port, a tube delivering air for culture aeration, a port for a spectrophotometer, and a resistance-based liquid level sensor. A distinguishing feature of our setup are custom-made syringe pumps and valves which enable us to precisely (<1%) control the volume of injected and pumped-out media. Each bottle is equipped with a miniature spectrophotometer to measure the optical density. This is achieved by aspirating each culture every 10 s into a specially designed glass cuvette at the top of the bottle, passing light from a narrow-beam LED (wavelength = 600 nm) through it, and measuring the intensity of the incoming and transmitted light.

All cultures are connected to an air pump for aeration, and stirred vigorously using magnetic beads at the bottom of each bottle. An additional sample tube with a syringe at the end is used for inoculation and sampling. The culture bottles as well as the pumps and the electronics are placed in a temperature-controlled incubator with fans that ensure uniform temperature of all cultures.

The bioreactor is controlled by a PC using custom-made software that monitors the optical density, temperature, and controls the injection of LB and antibiotics. Dilution occurs whenever the OD reaches OD = 0.1 or after 30mins, whichever happens first. Each dilution replaces 25% of the culture volume with fresh medium. This guarantees that only cells with growth rate g>(−log0.75)/0.5=0.58 h^-1^ (doubling time < 72 min) can persist in the culture. The software automatically calculates how much antibiotic to add to achieve a pre-determined, time-dependent concentration in the culture. The actual concentration of the antibiotic is not monitored.

Prior to the experiment, culture bottles are removed from the system and autoclaved, and (after re-attaching the bottles) the system is flushed with bleaching agent (PRESEPT, 5 g / 1 L water) and three times with media to dilute out the bleach. This approach effectively sterilizes the equipment (no growth over many days in test runs with no inoculum). The incubator is switched on and, after the system has reached 37°C, each culture is inoculated with 1ml of bacterial suspension.

### Evolution experiments

Overnight cultures of *E. coli* AD30 and EEL02 were diluted 1:200 in 10mL each of fresh medium and pre-grown shaking at 37°C for 1 h before being mixed 1:1 and 1mL of this mixture was injected into each bottle. The inoculum was also diluted ${\text{1}:\text{1}\text{0}}^{\text{4}}$ into PBS and plated or spotted onto LB agar (100 μL/plate) and inoculated overnight at 37°C for CFU counts. The software was set to dilute each culture when OD600 of 0.1 was reached, or every 30 min. In the long-term experiments the antibiotic was added 2 hours after the optical density reached OD = 0.1 for the first time, and then maintained at the same concentration throughout the rest of the experiment. At the end of long-term experiments, all cultures where resistant regrowth had occurred were sampled by diluting the culture 1:${\text{1}\text{0}}^{\text{4}}$ in PBS and spotted or plated on LB agar for CFU counts (100 μL/plate). A sample of each resistant population was also frozen in –80C in 15% glycerol. In the short-term experiments in which pre-antibiotic resistant mutants were counted on antibiotic-agar plates, the experiments were stopped approximately 2 h after the optical density reached OD = 0.1 for the first time. The exact stop times differed from 2 hours, because the different replicate cultures reached OD = 0.1 at slightly different times but had to be stopped at the same time due to the design of the turbidostat setup. The entire cultures were poured into Falcon tubes kept on ice to prevent further cell division before plating. A small sample (10 μL) from each culture was diluted ${\text{1}:\text{1}\text{0}}^{\text{4}}$ into PBS before being spotted onto LB agar for CFU count and incubated at 37°C overnight, and the rest of the culture were centrifuged at 4C, 4700 RPM for 45 min. Most of the supernatant was carefully poured off and the pellet was resuspended in the remaining medium. The resuspended pellet was plated on LB agar plates containing the antibiotic used for selection. The plates were then incubated at 37°C for 48 hours to allow for growth of all resistant mutants present in the culture.

“Standing variation” experiments used a 1:1000 mixture (v/v) of overnight cultures of resistant:sensitive bacteria, pre-grown outside the bioreactor to make sure that the resistant culture as well as the sensitive wild type were growing exponentially upon inoculation, as confirmed by measuring OD over time. To get the actual initial ratio of resistant to sensitive cells, the inoculums were diluted 10x and spotted onto LB agar with the antibiotic for resistant cell count, and diluted $\text{1}:{\text{1}\text{0}}^{\text{4}}$ and spotted onto LB agar for total cell count, and then incubated overnight at 37°C.

To measure the growth rates of the resistant mutants in the bioreactor in the absence and presence of the antibiotic, each identified allele was grown overnight, diluted 1:200 in fresh LB and pre-grown for 1 h shaking at 37°C. 1 mL was then inoculated into the bioreactor. The antibiotic relevant for the resistance was added 2 h after the optical density reached OD = 0.1. In the cases where the alleles grew too slowly in the bioreactor, the time between dilutions was changed to 2 h instead of 30 min to enable the slow-growing alleles to persist in the culture.

### Fluctuation tests

A single bacterial colony was picked and inoculated in 1 mL of LB medium and grown overnight to stationary phase. This stationary culture was diluted 10^6^ times and cultured again for 24 hours to reach the stationary phase. This culture was diluted again 10^6^ times, divided into many small cultures. A small volume of the diluted culture was also plated onto non-selective agar to estimate the initial number of colony-forming units (CFUs). All the replicate small cultures were again incubated at 37°C for 24 hours, to ensure they reached stationary phase. Then a few cultures were diluted 10^6^ times and plated on LB agar plates to estimate the final density of cells. The remaining cultures were plated onto selective agar plates and incubated for 48 h, and the number of CFUs was counted. All results can be found in S2 Table.

### Bacterial strains

We used a derivative of the *E. coli* K12 strain MG1655 with a *fimA* deletion (AD30) [30], and an isogenic strain with constitutive YFP expression and chloramphenicol resistance (EEL02). The *fimA* deletion prevents biofilm formation in the bioreactor (as opposed to MG1655), probably due to the lack of functional fimbriae. The cassette with constitutive YFP expression and chloramphenicol resistance were moved from strain RJA002 [50] using P1 transduction [51]. In the STR experiment with an increased mutator rate we used two isogenic mutator strains where *mutS* was deleted using plasmid mediated gene replacement [52], using the following primers to amplify the upstream and downstream regions of the *mutS* gene: mutSup_fwd; AAAAACTGCAGAAACAACGCCTCGTAATGCT, mutSup_rev; CGGGAATTGTTAGGGGTTATGTCCGGT, mutSdown_fwd: GGACATAACCCCTAACAATTCCCGATA and mutSdown_rev: AAAAAGTCGACCACCGCTGGCAAGCCACATA, and using crossover PCR to anneal the regions before ligation into the pTOF24 plasmid used for the gene deletion. We deleted *mutS* in AD30 and an isogenic strain (EEL03) with constitutive CFP expression and ampicillin resistance, the cassette with CFP and ampicillin resistance were moved from strain RJA003 [50], using P1 transduction [51].

### Growth conditions

All experiments were performed in LB broth (Miller), at 37°C, and on LB (miller) agar plates with 1.5% agar-agar. Antibiotic stocks were prepared as follows; 50 mg/ml rifampicin in DMSO, 10 mg/ml ciprofloxacin hydrochloride in water, and 100 mg/ml streptomycin in water. We used concentrations of 100 μg/ml of rifampicin and streptomycin and 100 ng/ml ciprofloxacin in all experiments.

### Whole genome sequencing to identify resistance conferring mutations

Chromosomal DNA was extracted from each clone using the Monarch Genomic DNA Purification Kit (New England Biolabs). DNA libraries were prepared using the Rapid Sequencing DNA Kit with 96 barcodes (SQK-RBK110.96 or SQK-RBK114.96). The libraries were sequenced using the MinION Mk1B device (Oxford Nanopore), with R9.4.1 or R10.4.01 flow cells. The sequence data for all sequenced alleles has been deposited in the European Nucleotide Archive at EMBL-EBI under accession number PRJEB86481.

Basecalling, barcode splitting and aligning the resulting FASTQ files to the reference genome (*Escherichia coli* str. K-12 substr. MG1655, reference genome assembly GCF_000005845.2_ASM584v2) was done using guppy (Oxford Nanopore Technologies plc., version 6.5.7 + ca6d6af). Variant calling was performed with freebayes (v1.3.6) [53] using the default parameters, except that the minimum coverage was set to 5. To detect large deletions, we used delly (v1.1.5) [54] as well as visual inspection of genome coverage using Geneious (https://www.geneious.com/). Since Nanopore sequencing is known to generate many artefacts, especially in regions rich in k-mers [55], we treated all detected variants containing k-mers with k>3 as artefacts. To further reduce the probability of artefacts when detecting SNPs, we focused only on genes with known relevance for RIF, CIP, and STR resistance: *gyrA*, *gyrB*, *parC*, *marR*, *marA*, *marB*, *rpsL*, *rpoB*. Occasional SNPs detected in other regions showed no consistency between the altered region and the antibiotic used, and did not repeat in samples treated in the same way. We therefore considered such SNPs to represent sequencing artefacts.

### Bioreactor data analysis

All analysis has been done using Wolfram Mathematica notebooks.

#### Growth rate.

For each OD-vs-time curve we obtained a sequence of growth rates $\left\{g_{i}\right\}$ corresponding to time periods between consecutive dilutions by fitting a piece-wise exponential function with a background correction


$$\begin{array}{c} \text{OD}=c+\text{exp}(g\left(t-t_{\text{2}}\right))\left\{ \begin{array}{c} @la,\;ift<t_{\text{2}}\\ af,\;ift\geq t_{\text{2}}\; \end{array}\right. \end{array}$$

to every two consecutive dilution cycles $t_{\text{1}}\to t_{\text{2}}$ and $t_{\text{2}}\to t_{\text{3}}$. In the equation above, a,b,c are three free parameters of the fit. This approach enabled a more unambiguous estimate of the background correction c than would be possible by fitting a single exponential to one cycle. The approach was tested using synthetic data, i.e., we run the simple birth-mutation-dilution model with a known growth rate to generate the OD vs time data, and confirmed that the above fitting procedure recovered the growth rate. The accurate determination of the dilution factor f=0.75 plays an important role here, hence we performed experiments with the methylene blue dye subject to periodic dilutions to make sure the dilution factor was 0.75 in each culture as assumed above.

#### Growth rate of the sensitive population.

We took all $\left\{g_{i}\right\}$ that came from dilution cycles before the antibiotic and in which the minimum OD was at least 0.05.

#### Growth rate of the resistant population.

We took all $\left\{g_{i}\right\}$ from the regrowth phase which had the minimum OD of at least 0.05.

#### Initial optical density at 𝐭=0.

Since the culture was initially very diluted and measuring its OD would not be accurate, we obtained ${\text{OD}}_{\text{initial}}$ by extrapolating backwards from the growth phase with OD > 0.075 using the growth rate determined as described above, and assuming exponential growth since the beginning of the experiment. This gave ${\text{OD}}_{\text{initial}}$ values between 0.002 and 0.005, in agreement with what was expected based on the volume and density of the inoculum as well as the direct measurement of ${\text{OD}}_{\text{initial}}$ at t=0.

#### Conversion factor Q between the optical density and bacterial density.

To establish the proportionality coefficient between bacterial and optical density (no. of cells/ml per OD = 1), we collected samples from a single bioreactor run (4 cultures) corresponding to the steady-state exponential growth phase (about 5000 s since the OD reaching OD = 0.1 for the first time). The samples were diluted $\text{1}:{\text{1}\text{0}}^{\text{4}}$ times and plated (100 ml) on LB agar plates (two replicates for each culture). To maximize plating efficiency, we spot-plated each 100 ml, i.e., we touched the agar with a pipette tip at tens of positions, each time expressing only a small volume of the 100 ml total volume. We verified by comparing colony counts with counting bacteria from the same cultures under the microscope that this technique was much more reliable than expressing the entire volume at once and spreading it using a sterile spreader. The corresponding optical densities and cell counts were (OD, CFUs) = [[0.0823, 196 + 176], [0.0929, 155 + 199], [0.0866, 216 + 194], [0.0939, 206 + 183]], from which we calculated the conversion factor to be $\text{2}.\text{1}\text{5}\times {\text{1}\text{0}}^{\text{8}}$ bacteria/unit OD. In the simulations, we used a rounded-down number $Q^{-\text{1}}=\text{2}\times {\text{1}\text{0}}^{\text{8}}$ bacteria/OD.

### Mathematical modelling

We used the generalised version of the birth process [23] with two species: sensitive and resistant cells, and with mutations. Sensitive cells divide with rate $g_{\text{S}}$ in the absence of antibiotics, and do not replicate when the antibiotic is present. Replication results in a new sensitive cell with probability 1−μ, or with a resistant cell with probability μ. Resistant cells divide with rate $g_{\text{R}}$ (same in the presence/absence of the antibiotic).

To simulate bioreactor experiments, the number of sensitive bacteria $n_{\text{S}}$ is set to the number calculated from the initial optical density as explained above, and the number of resistant bacteria $n_{\text{R}}$ is set to zero. Growth is simulated using an approximate stochastic simulation protocol - the “tau leaping algorithm” [56] with a fixed time step of 1/64 h. Dilution is implemented by generating new $n_{\text{S}}^{\prime },r_{\text{R}}^{\prime }$ from binomial distributions with means $n_{\text{S}},r_{\text{R}}$ and the success rate f=0.75 equals the fraction of the volume retained after a single dilution step.

To model filamentation (CIP) or a more general delayed response to the antibiotic (STR), we used a time-varying conversion factor between the number of cells and the optical density:


$$\begin{array}{c} \text{OD}=\text{O}{\text{D}}_{\text{R}}+\text{O}{\text{D}}_{\text{S}} \end{array}$$

$$\begin{array}{c} \text{O}{\text{D}}_{\text{R}}=Q_{\text{R}}n_{\text{R}} \end{array}$$

$$\begin{array}{c} \text{O}{\text{D}}_{\text{S}}=Q_{\text{S}}n_{\text{S}} \end{array}$$

$$\begin{array}{c} \frac{\text{d}\left(Q_{\text{S}}\right)}{\text{d}t}=g_{\text{S}}\left(\text{1}-{\left(\frac{t-t_{\text{0}}}{\tau }\right)}^{\text{2}}\right) \end{array}$$

where $Q_{S}(\text{0})=Q_{R}=\text{1}/(\text{2}\times {\text{1}\text{0}}^{\text{7}}$), and τ is set to 2 h for CIP and 0.66 h for STR to account for the observed time delay (S2 Fig).

To model different mutation probabilities, we first estimated the relative probability $P_{\text{allele }i}$ of occurrence of different SNPs found in our evolutionary experiments. We then modified the model so that, while the total mutation probability to resistance was still μ, a specific allele i would be selected with probability $\mu P_{\text{allele }i}$.

To account for RIF contribution to the OD, we modelled RIF and its decay product rifampicin quinone (RIFO) using the following differential equations


$$\begin{array}{c} \frac{\text{d}c_{\text{RIF}}}{\text{d}t}=-\lambda c_{\text{RIF}} \end{array}$$

$$\begin{array}{c} \frac{\text{d}c_{\text{RIFO}}}{\text{d}t}=\lambda c_{\text{RIF}} \end{array}$$

$$\begin{array}{c} \text{OD}=\text{O}{\text{D}}_{\text{bacteria}}+c_{\text{RIF}}+\beta c_{\text{RIFO}} \end{array}$$

Dilution was modelled as follows: $c_{\text{RIF}}\to fc_{\text{RIF}}+(\text{1}-f)c_{\text{0},\text{ RIF}},c_{\text{RIFO}}\to fc_{\text{RIFO}}$

The model has three parameters: $c_{\text{0},\text{RIF}}$ which describes the theoretical OD contribution if the entire culture was replaced by RIF from the reservoir, β which describes by how much more the decay product contributes to the OD compared to RIF, and λ which is the RIF decay rate. We first estimated β by fitting this model to an experiment in which we added RIF to the culture in the same way as in the long-term evolutionary experiment but without any bacteria present. The λ rate could in principle depend on the chemical composition of the culture, in particular its pH, and temperature. Similarly, $c_{\text{0},\text{RIF}}$ could differ for different experiments since RIF may have decayed already in the reservoir to an unknown extent. This is not a problem from the RIF antimicrobial activity standpoint because the RIF quinone is also active against bacteria, but it means each replicate RIF experiment may have a slightly different background OD after the first RIF injection. Therefore, to obtain λ and $c_{\text{0},\text{RIF}}$ we fitted the RIF decay model to experimental long-term OD vs time curves in the regime in which the curve was close to zero (almost no bacteria in the culture), and the only contribution to OD was due to RIF and RIFO. This gave the value of λ between 0.15 and 0.2 h^-1^ for different experiments, and $c_{\text{0},\text{RIF}}$ between 0.016 and 0.032. These values were then used to subtract the RIF contribution each individual OD-vs-time curve.

All models have been implemented in C++.

#### Probability distributions from the model.

To obtain the regrowth probability, the distribution of regrowth times, and FLAS, each model was simulated 1000 times for each of the experimental replicates, using experimentally determined growth rates (pre- and post-antibiotic if available, i.e., if regrowth occurred) from that replicate. If a given replicate showed no regrowth, the regrowth (resistant) growth rate was randomly assigned from the remaining replicates.

To obtain the error bars in Fig 2E, we used confidence intervals of the estimated regrowth probability. Assuming the number of experiments showing regrowth (“success”) followed the binomial distribution, the confidence interval for $P_{\text{regrowth}}$ was calculated using the Wilson score interval:


$$\begin{array}{c} P_{\text{regrowth}}=\frac{m+\frac{z^{\text{2}}}{\text{2}}}{n+z^{\text{2}}}\pm \frac{z}{n+z^{\text{2}}}\;\sqrt{\frac{m(n-m)}{n}+\frac{z^{\text{2}}}{\text{4}}} \end{array}$$

We used z=1 for the 68% “one sigma” confidence interval.

#### Statistical tests.

To compare experimental data and model predictions we used Kolmogorov-Smirnov test (*KolmogorovSmirnovTest* in Wolfram Mathematica) for quantities such as the regrowth time, the fraction of the least-abundant strain, and the number of resistant mutants. We used Fisher exact test (our own implementation in Mathematica) for the regrowth probability. All these tests return a p-value which is what we use to determine whether a model is a good fit to the data (p-value above 0.1) or not.

#### The mutation probability from fluctuation test data.

We used the Lea and Coulson model (birth process with mutations) [23] to determine μ from the fluctuation test data. Specifically, we used P(m;μ), the probability of finding m mutants assuming the mutation probability μ per replication, to calculate the posterior probability distribution for a vector of N measurements $\left\{m_{\text{1}},...,m_{N}\right\}$:


$$\begin{array}{c} \text{ln}P(\mu )=\sum_{i}^{N}\text{ln}P(m_{i};\mu ) \end{array}$$

and find a value of μ which maximized this probability. To calculate P(m;μ) we used the recursive relation [35].

#### Optimal mutation probability μ from the mutant distribution data.

We searched for a value of μ for which the p-value of the Kolmogorov-Smirnov test comparing the experimental and simulated distributions of the number of mutants before the antibiotic was maximized. We wrote a Wolfram Mathematica function which returned this p-value using a simulated distribution from 300 independent simulations, for a given value of μ, and used this in a minimization routine (“golden-section search”), suitably bracketing the optimum μ to a biologically-realistic range.

#### Optimal phenotypic delay parameters.

We proceeded similarly to when finding the optimal μ, but this time we maximized the expression:


$$\begin{array}{c} p_{\text{1}}+\text{100 min}\left(p_{\text{2}}-\text{0}.\text{1},\text{0}\right)+\text{100 min}\left(p_{\text{3}}-\text{0}.\text{1},\text{0}\right) \end{array}$$

where $p_{\text{1}},p_{\text{2}},p_{\text{3}}$ are p-values of KS tests comparing the simulated and experimental observables: the regrowth probability, the distribution of regrowth times, and the distribution of the least-frequent allele. The function significantly penalized any p-value larger than 0.1, thus forcing such parameter values that would lead to all p-values below 0.1 if at all possible. Since the optimization problem was now two-dimensional and the function could in principle have many local minima, we used a brute-force approach: we calculated the fitting function for a range of μ and ω values regularly spaced on a grid, with a suitably small step in each direction, and biologically realistic boundaries. See the Mathematica code for more details.

#### The risk of overfitting.

None of the new models has more than two free parameters (or three if the mutation rate — occasionally fitted to mutant distribution data — is included). We compare each model against three observables: the regrowth probability, the distribution of regrowth times, and the fraction of the least abundant strain. Since two of these are full distributions rather than single values, the fitting procedure can still fail because the number of fitted parameters is smaller than the number of observables. Some models (e.g., the multiple allele model) may seem to involve additional parameters, but these parameters are always determined independently rather than fitted to the same data the model aims to reproduce. As a result, the risk of overfitting remains low.

### Data and code

All code (C++ code for simulating the model, Mathematica notebooks for data analysis and plotting) and bioreactor data are available on GitHub: https://github.com/Dioscuri-Centre/AMR_bioreactor. DNA sequences are available from the European Nucleotide Archive at EMBL-EBI under accession number PRJEB86481.

## Supporting information

S1 Fig Rifampicin contribution to the optical density. (A) Absorption spectrum of freshly prepared rifampicin (100 ug/ml) in LB. (B) A range of the same spectrum magnified to show absorption at 600 nm - the wavelength used in the spectrophotometer to measure the optical density. RIF is thus expected to contribute about 0.02 to the total OD, which is about 20% of the maximum expected OD in our bioreactor experiments. (C) OD versus time for a bioreactor experiment in which rifampicin was injected at t = 2.5 h and added during subsequent dilution steps to maintain the concentration of 100 ug/ml. The measured optical density changes in time due to rifampicin slowly converted to a more absorbing product (rifampicin quinone) with rate constant lambda. Fitting the model to data (3 replicates; only one shown here for clarity) gives lambda = 0.5 h^-1^ and the RIF-quinone contribution to the OD = 3.3 times the contribution of pure RIF. The decay rate lambda is different in the presence of bacteria (lambda = 0.2 h^-1^, obtained from model fitting to long-term evolutionary experiments, see Methods); this rate was used to subtract RIF contribution from OD vs time curves in Fig 2.
(DOCX)

S2 Fig Comparison of the response times for CIP, STR, and RIF. Plots show the growth rate before and after the antibiotic (vertical red line at t = 0).
(DOCX)

S3 Fig The model with time-varying cell-density to optical density conversion factor, for CIP and STR. Black = experimental curves. Light green = model with constant conversion factor (as in Fig 2C). Dark green = model with time-dependent factor.
(DOCX)

S4 Fig Fluorescent images of individual cells before and after antibiotic exposure. **(A-B)** Cells before STR (A) and CIP (B) exposure. **(C)** Cells 100 min after the exposure to STR. **(D)** Cells 100 min after the exposure to CIP. Yellow-fluorescent strain EEL02 was used in each case, and the white scale bar = 10 μm. Imaging settings: 40x NA 0.9 air objective, exposure time: 300 ms, camera: Andor Zyla 4.2+, filter: FIT-C, excitation lamp: CoolLED pE300 at 20% power. Bacteria were deposited on a glass slide and immediately covered with a cover slip #1. Images of well-focused individual cells were cropped, rotated 90° whenever required to make the cells better aligned horizontally, and assembled into a collage. Only a fraction of all imaged cells is shown for each condition.
(DOCX)

S5 Fig The simple model correctly reproduces resistant regrowth in a standing variation experiment. Replicates of OD versus time for bioreactor experiments in which a sensitive population spiked-in with a small number of resistant mutant cells was exposed to RIF, CIP, and STR. Black = experiment, green = model prediction (1000 simulations presented as the probability heatmap). The red line marks the beginning of antibiotic exposure. Mutants used and their initial fractions (determined by plating): RIF: *rpoB* D516Y, ratio 1:1000; CIP: *gyrA* S83L, ratio 1:1000; STR: *rpsL* K43T, ratio 1:1660. The right-most column shows regrowth time distributions and the p-values for KS tests comparing the experimental and predicted distributions.
(DOCX)

S6 Fig Additional models for RIF. Rows of plots show: the probability of resistant regrowth, the time to regrowth, the fraction of the least abundant strain at the end of resistant regrowth, and the distribution of the number of resistant mutants prior to antibiotic exposure. Grey and green histograms are the experimental and simulated data, respectively.
(DOCX)

S7 Fig Additional models for CIP. Rows of plots show: the probability of resistant regrowth, the time to regrowth, the fraction of the least abundant strain at the end of resistant regrowth, and the distribution of the number of resistant mutants prior to antibiotic exposure. Grey and green histograms are the experimental and simulated data, respectively.
(DOCX)

S1 Table A list of mutations detected in samples from long- and short-term experiments. (XLSX)

S2 Table Fluctuation tests results for different strains and antibiotics. (XLSX)
