# Supplementary material for: Simple birth-death-mutation models predict some—but not all—aspects of the experimental evolution of antibiotic resistance
Source: PLoS Comput Biol. 2026 Aug 28;22(8):e1014666. doi: 10.1371/journal.pcbi.1014666 (PMC13552961; doi:10.1371/journal.pcbi.1014666)
Supplement: Supplementary file 1 [file pcbi.1014666.s001.docx]

**S1 Fig. Rifampicin contribution to the optical density.** (A) Absorption spectrum of freshly prepared rifampicin (100 ug/ml) in LB. (B) A range of the same spectrum magnified to show absorption at 600 nm - the wavelength used in the spectrophotometer to measure the optical density. RIF is thus expected to contribute about 0.02 to the total OD, which is about 20% of the maximum expected OD in our bioreactor experiments. (C) OD versus time for a bioreactor experiment in which rifampicin was injected at t=2.5 h and added during subsequent dilution steps to maintain the concentration of 100 ug/ml. The measured optical density changes in time due to rifampicin slowly converted to a more absorbing product (rifampicin quinone) with rate constant lambda. Fitting the model to data (3 replicates; only one shown here for clarity) gives lambda=0.5 h^-1^ and the RIF-quinone contribution to the OD = 3.3 times the contribution of pure RIF. The decay rate lambda is different in the presence of bacteria (lambda=0.2 h^-1^, obtained from model fitting to long-term evolutionary experiments, see Methods); this rate was used to subtract RIF contribution from OD vs time curves in Fig. 2.
