# Supplementary material for: Simple birth-death-mutation models predict some—but not all—aspects of the experimental evolution of antibiotic resistance
Source: PLoS Comput Biol. 2026 Aug 28;22(8):e1014666. doi: 10.1371/journal.pcbi.1014666 (PMC13552961; doi:10.1371/journal.pcbi.1014666)
Supplement: Supplementary file 2 [file pcbi.1014666.s002.docx]

**S2 Fig. Comparison of the response times for CIP, STR, and RIF**. Plots show the growth rate before and after the antibiotic (vertical red line at t=0).
