# Supplementary material for: Simple birth-death-mutation models predict some—but not all—aspects of the experimental evolution of antibiotic resistance
Source: PLoS Comput Biol. 2026 Aug 28;22(8):e1014666. doi: 10.1371/journal.pcbi.1014666 (PMC13552961; doi:10.1371/journal.pcbi.1014666)
Supplement: Supplementary file 3 [file pcbi.1014666.s003.docx]

**S3 Fig. The model with time-varying cell-density to optical density conversion factor, for CIP and STR.** Black = experimental curves. Light green = model with constant conversion factor (as in Fig. 2C). Dark green = model with time-dependent factor.
