# Supplementary material for: Simple birth-death-mutation models predict some—but not all—aspects of the experimental evolution of antibiotic resistance
Source: PLoS Comput Biol. 2026 Aug 28;22(8):e1014666. doi: 10.1371/journal.pcbi.1014666 (PMC13552961; doi:10.1371/journal.pcbi.1014666)
Supplement: Supplementary file 4 [file pcbi.1014666.s004.docx]

**S4 Fig. Fluorescent images of individual cells before and after antibiotic exposure. (A-B)** Cells before STR (A) and CIP (B) exposure. **(C)** Cells 100 min after the exposure to STR. **(D)** Cells 100 min after the exposure to CIP. Yellow-fluorescent strain EEL02 was used in each case, and the white scale bar = 10 mm. Imaging settings: 40x NA 0.9 air objective, exposure time: 300 ms, camera: Andor Zyla 4.2+, filter: FIT-C, excitation lamp: CoolLED pE300 at 20% power. Bacteria were deposited on a glass slide and immediately covered with a cover slip #1. Images of well-focused individual cells were cropped, rotated 90° whenever required to make the cells better aligned horizontally, and assembled into a collage. Only a fraction of all imaged cells is shown for each condition.
