# Supplementary material for: Simple birth-death-mutation models predict some—but not all—aspects of the experimental evolution of antibiotic resistance
Source: PLoS Comput Biol. 2026 Aug 28;22(8):e1014666. doi: 10.1371/journal.pcbi.1014666 (PMC13552961; doi:10.1371/journal.pcbi.1014666)
Supplement: Supplementary file 5 [file pcbi.1014666.s005.docx]

**S5 Fig. The simple model correctly reproduces resistant regrowth in a standing variation experiment.** Replicates of OD versus time for bioreactor experiments in which a sensitive population spiked-in with a small number of resistant mutant cells was exposed to RIF, CIP, and STR. Black = experiment, green = model prediction (1000 simulations presented as the probability heatmap). The red line marks the beginning of antibiotic exposure. Mutants used and their initial fractions (determined by plating): RIF: *rpoB* D516Y, ratio 1:1000; CIP: *gyrA* S83L, ratio 1:1000; STR: *rpsL* K43T, ratio 1:1660. The right-most column shows regrowth time distributions and the p-values for KS tests comparing the experimental and predicted distributions.
