# Supplementary material for: Simple birth-death-mutation models predict some—but not all—aspects of the experimental evolution of antibiotic resistance
Source: PLoS Comput Biol. 2026 Aug 28;22(8):e1014666. doi: 10.1371/journal.pcbi.1014666 (PMC13552961; doi:10.1371/journal.pcbi.1014666)
Supplement: Supplementary file 7 [file pcbi.1014666.s007.docx]

**S7 Fig. Additional models for CIP.** Rows of plots show: the probability of resistant regrowth, the time to regrowth, the fraction of the least abundant strain at the end of resistant regrowth, and the distribution of the number of resistant mutants prior to antibiotic exposure. Grey and green histograms are the experimental and simulated data, respectively.
