# Supplementary material for: Simple birth-death-mutation models predict some—but not all—aspects of the experimental evolution of antibiotic resistance
Source: PLoS Comput Biol. 2026 Aug 28;22(8):e1014666. doi: 10.1371/journal.pcbi.1014666 (PMC13552961; doi:10.1371/journal.pcbi.1014666)
Supplement: Supplementary file 8 [file pcbi.1014666.s011.docx]

We thank all three Reviewers for their constructive comments. Please find below a response to each comment. Reviewer’s original comments are in black, our response is in blue.

**Reviewer #1:** The manuscript presents clear analyses and reports interesting results. I appreciate the transparent exposition and the careful presentation of simulations and comparisons. I appreciate the manuscript’s coupling of experiments and theory.

Major comments:

- While the findings are interesting, it remains unclear how broadly they generalize or how they should be used in practice. The take-home message that birth-death-mutation (BDM) models can predict some aspects of resistance evolution but not others, is somewhat unsurprising. It would help if the authors articulated what is novel and useful here, and concluded with concrete guidance on use: for example, should BDM models be treated as null models for baseline predictions, stress-tests/sanity checks, or quick screening tools before deploying richer frameworks?

There are several novel and useful findings here:

- The BDM model fails in different ways for different antibiotics, each time giving an insight into what factors are important when modelling resistance. Being aware of these factors is important for future, quantitative and predictive models of resistance evolution.
- The paper states what we think should be the baseline for predictive models, and specifies what magnitude deviations one may expect when neglecting extensions of BDM.
- Lastly, BDM models are popular models often used (and abused) in various context. Our paper clearly shows the limitations of these models. The Reviewer is correct that this is somewhat unsurprising but we have never seen it articulated this clearly.

We have added these points to several paragraphs of the discussion section, including a new subsection “Broader implications”.

- The introduction needs more context. There are several other models that have been employed to study resistance evolution, for example, branching processes, models involving division times between cells, diffusion models, consumer-resource models, etc. It may also help to note common modeling extensions that are relevant in practice: PK/PD coupling, non-genetic versus genetic resistance, and drug-specific mechanisms. Also, it would be useful to list the assumptions behind BDM models (e.g., well-mixed population, no epistasis, no competition, absence of spatial structure, etc.).

We appreciate these comments; we have added a clear statement of the assumptions behind the models discussed here to “Introduction”. On the other hand, it is not our purpose to write a comprehensive review, which is why we have limited ourselves only to discussing minimal models that account for growth and mutations, which is the focus of our work.

- What is the reason for using the tau-leaping algorithm in your study? My guess is that with exact algorithms (such as the Gillespie algorithm), the simulations are very costly at the population sizes considered. While the tau-leaping algorithm is a useful tool that speeds up simulations, its approximations can introduce bias. Part of the discrepancies between theory and data that you observe could come from using this algorithm. Have the authors validated this algorithm for the cases studied? Including a small-scale benchmark against the Gillespie algorithm (on subsets where exact simulation is feasible) would strengthen confidence in your results.

The Reviewer is correct that an exact algorithm would be too costly to simulate. However, in the limit of $dt\to0$, the algorithm we use becomes exact. We have confirmed that the approximate algorithm is accurate enough for small but finite $dt$. To do this, we simulated the same process (the simple model for CIP, no filamentation, one resistant allele, no lag) with different fixed $dt$’s. The figure below compares the probability of regrowth and the distribution of time to regrowth for four different dt’s:

|  | $\boldsymbol{dt=}\boldsymbol{2}^{\boldsymbol{-5}}$ | $\boldsymbol{dt=}\boldsymbol{2}^{\boldsymbol{-6}}$ | $\boldsymbol{dt=}\boldsymbol{2}^{\boldsymbol{-7}}$ | $\boldsymbol{dt=}\boldsymbol{2}^{\boldsymbol{-10}}$ |
| --- | --- | --- | --- | --- |
| $P_{\mathrm{regrowth}}$ 95% confidence interval | 0.609 … 0.620 | 0.599 … 0.609 | 0.597 … 0.607 | 0.597 … 0.608 |
| Mean $t_{\mathrm{regrowth}}-t_{0}$ +/- SEM | 16.79 +/- 0.03 | 16.85 +/- 0.03 | 16.80 +/- 0.03 | 16.78 +/- 0.03 |

It is evident that, as $dt$ decreases, the results converge to the limit case $dt\to0$. Our simulations in the manuscript use the value $dt=2^{-6}$ which, as evident from the above table, differs from a much smaller $dt=2^{-10}$ by less than 1%, which is well below any experiment-related variability.

- A weak point of this study is that modifications to the model are introduced only after observing discrepancies. The modifications are justified to some extent, but several different modifications could have been considered to produce similar observables. Adding them post-hoc can fit data but weakens generality. I understand the main purpose of this paper is not to explain the mechanisms behind, but the paper could benefit from discussing these points. Besides, adding extra modifications has the risk of overfitting data. Perhaps the author could comment on that as well. If possible, clarifying which modifications are necessary versus merely sufficient would help readers gauge portability to new datasets.

As the Reviewer notes, our aim is not to explain the exact mechanism of resistance evolution but to show where a simple model fails for each antibiotic, and find a minimal model that is biologically plausible and able to reproduce the data. We do this step by step, extending the models by adding processes with at most two free parameters (for example, $\omega$ and $q$). Some models (e.g., the multiple allele model) may look like they have more parameters, but these parameters have been determined independently and not fitted to data which the model is supposed to reproduce. Given that we fit the model to three observables (regrowth probability, regrowth time distribution, the fraction of the least abundant strain), two of which are probability distributions and not single numbers, the fitting procedure could still fail as the model has fewer parameters than the number of observables. We therefore do not think the data are overfitted.

We commented on this in a new subsection “The risk of overfitting” in Methods.

Minor comments:

- The paper is generally very clear, but Figure 4 is visually crowded. Consider increasing the gap between panels and increasing the font size. I recommend also adding an x-axis to the second-row panels.

We have added the symbols “E” for experimental data and “M” for the model to the second row of panels, and explained the symbols in the caption. We have added more vertical space between the panels, and moved $\mu$ values to the top row, below graphical representations of the models.

- Consider improving the notation in your equations. Perhaps a piecewise notation with intervals could improve their readability.

We have modified the expressions in sections “Growth rate” and “Optimal phenotypic delay parameters”

**Reviewer #2:** Summary:

In this paper, the authors investigate evolution of resistance to three antibiotics in bioreactor cultures that allow for continuous exponential growth. The authors attempt to rigorously test how well a type of birth-death-mutation process can quantitatively explain the observed experimental results by looking at some more nuanced observables such as regrowth time distributions. While numerical simulations of the mathematical model largely match with the observed trajectories, the authors show that some quantitative discrepancies arise depending on the used antibiotic and then present some extensions to the model that can better explain these deviations.

By reading the paper it is apparent that this work is of high quality. Although there is limited new computational or mathematical advances, the experimental setup is quite impressive, and I agree with the authors that biology needs more rigorous application of quantitative methods. While I am not sure I fully agree with all the reached conclusions and the overall framing of the paper (please see more detailed comments below), I think it is in principle acceptable to PLOS Computational Biology without needing any major changes.

Comments:

1. The codes cannot be accessed from the provided link. Note that this journal requires all the materials to be made available for review.

The repository was made a private repository by mistake. We have now changed it to a public repository. We apologize for overlooking this in the initial submission.

2. The paper is written in a way that presents the birth-death-mutation process as an agreed “null model” that the authors then aim to reject. While this is certainly a refreshing take in this context, I am not convinced that this is the case. Firstly, because there are many different flavours and more subtle versions of such models, but also other simple models that could be used that have been developed especially in the evolutionary rescue literature. Thus, I am not sure what the quite small discrepancies of this particular model ultimately tell us. Rigorous hypothesis testing first needs a clear null model or proposed hypothesis/mechanism, which I don’t think is very clear here. What is the precise assumption of the model that we can now reject?

We clearly state the research question in the introduction: “In this work we ask to what extent the birth-death-mutation mathematical model can quantitatively reproduce the evolution of antibiotic resistance in a bioreactor model of antibiotic treatment”, and subsequently address it by comparing various predictions of the model with experimental data. The discrepancies discovered (not necessary very small when one looks at absolute numbers, such as the probability of resistance) suggest what may be missing from the simple model, and we show that adding the missing components significantly improves the model’s agreement with data.

We stress that our aim here is not to reject a “null hypothesis” or to fully understand molecular mechanisms behind the evolution of resistance. Our aim is to show what level of accuracy one may expect when comparing simple models with experiments, and how small- but significant modifications improve the accuracy of the models. We believe that modelling of antibiotic resistance evolution has relied too heavily on simple models that provide only qualitative predictions. Here, we aim to advance this comparison by making it quantitative, showing that although the simple model fails in several respects, it can be readily modified to match the data. We argue that these modified models—rather than the basic birth–death model—should now be regarded as the appropriate “null models” in the field.

We have added some of these points to the discussion.

3. Continuing on this, my reading of the paper is that simple models, indeed simple birth-death-mutation models, can explain the data. The main difficulties are more related to the challenge of translating and inferring the model variables and parameters from the biological observables. Certainly, the authors haven’t shown that an even simpler model could not explain everything without needing too many modifications for each antibiotic. I think the main title is in this sense a bit misleading and would prefer the short title instead (“Testing simple models of antibiotic resistance evolution”).

We have changed the title to “Simple birth-death-mutation models predict some - but not all – aspects of the experimental evolution of antibiotic resistance”. We hope this revision makes the title more accurately reflect the content of the manuscript.
4. I think the observation that the inferred mutation rate from the fluctuation test is different from the apparent mutation rate in the bioreactor is one of the more interesting findings of the paper that could be expanded as this is likely important both biologically as well as in terms of thinking about the experiments. This could also be nicely linked to theory.

We have already discussed this in the section “The generation of CIP and STR resistant clones…” in the following paragraph: “This discrepancy suggests that the mutation probability that was measured in the fluctuation assays is not an accurate estimate of the actual mutation probability in the bioreactor. This could be due to the difference in growth conditions: in the bioreactor, cells are maintained in steady exponential growth whereas in the fluctuation assay they experience exponential growth followed by stationary phase.”

We hesitate to expand more on this, as the manuscript is already quite long and contains many results (e.g., different “failure modes” of the simplest model for different antibiotics) that are also interesting in our opinion.

5. Why is the number of replicates for each antibiotic different? Was some data excluded from the analysis?

The Reviewer is correct that some replicates were excluded due to problems noticed during- or post- experiments: contamination, hardware issues, control software crashing, mistakes during the set-up. We emphasize that these experiments – despite their apparent simplicity – were quite challenging because everything had to work perfectly for an experiment to produce quantitative, reliable data. It has taken several years to optimize the experimental design and make sure that problems would occurs with an acceptable rate (10% or less of all experiments) and, when they occurred, they could be detected and the corresponding data removed from the analysis.

6. How are the probability distributions for the regrowth times predicted by the model computed exactly in Fig. 2D? Their shape looks a bit weird to me, and given the simplicity of the model, some analytical results might also be possible.

To obtain the distribution of regrowth times, the corresponding model was simulated 1000 times for each of the experimental replicates, using experimentally determined growth rates (pre- and post-antibiotic if available, i.e., if regrowth occurred) from that replicate. If a given replicate showed no regrowth, the regrowth (resistant) growth rate was selected at random from the remaining replicates. This is why the distributions are not always unimodal; if there were few experiments to sample from (most prominent in the case of STR), the distribution would reflect the discreteness of the data set.

We have added this explanation to Methods (“Probability distributions from the model”).

7. In Fig. 2E, what is the meaning of the error bars? Doesn’t one simply compute one number for the empirical probability of regrowth from all the replicates?

Error bars in Fig. 2E represent confidence intervals of the estimated regrowth probability. Assuming the number $M$ of experiments showing regrowth (“success”) follows the binomial distribution with the number of trials $N$ and the success probability $P_{\mathrm{regrowth}}$, the estimate for $P_{\mathrm{regrowth}}=M/N$ is a random variable that has a certain distribution instead being a single number. The confidence interval for $P_{\mathrm{regrowth}}$ (the range of values around $P_{\mathrm{regrowth}}$ in which the true regrowth probability will fall with a given probability, say 68% for “one sigma” deviation) can be calculated using the Wilson score interval:

$${(m+1/2z^2)}/{(n+z^2)}\pm z/{(n+z^2)}Sqrt[m{(n-m)}/n+{z^2}/4]$$

where z=1 for the 68% “one sigma” confidence interval. This is what we assumed for Fig. 2E.

We have added a short explanation to the caption of Fig. 2, and a more detailed explanation to Methods (“Probability distributions from the model”).

**Reviewer #3:** The study by Lilja et al. investigates how mathematical models describing experimental evolution of antibiotic resistance can be made more accurate. The authors examine resistance evolution in a controlled bioreactor system, fitting model predictions to their empirical results. Overall, the manuscript is well-written, and the experiments appear technically sound. However, I have several major and minor concerns that should be addressed before publication.

Major Concerns

1) The manuscript demonstrates that including mechanistic components improves the quantitative match between model predictions and experimental measurements. However, it remains unclear what these refinements teach us about the evolution of resistance itself. Specifically, it is not shown whether these parameters, once included, actually alter the evolutionary outcome or trajectory in a meaningful way. As written, the narrative primarily demonstrates that including mechanistic realism improves predictive accuracy, which is interesting but an intuitive result. What is missing is a demonstration that these mechanisms affect the evolutionary dynamics, rather than merely helping the model fit the data post hoc. For example, does varying the phenotypic-lag parameter change the probability of resistance emergence, the distribution of resistant alleles, or the timing of regrowth in a way that yields new insight into RIF resistance evolution? Will it change evolutionary outcomes in experimental evolution studies using these antibiotics? Without such analyses, it is difficult to see how these additions reveal new principles about antibiotic action or resistance evolution. I encourage the authors to show how inferred parameters shape long-term evolutionary outcomes, or to explicitly discuss the conceptual advances arising from these refinements.

This is a fair point, and while many of the questions the Reviewer is asking can be answered based on statements already included in the manuscript, we have not done it explicitly. We have now added a discussion of how the new additions alter the behaviour of the basic growth-and-mutation model, as well as a new Figure 5.

2) While I appreciate the authors’ stepwise refinement of the mathematical models by incorporating mechanistically motivated features (e.g., filamentation for CIP, delayed initial response for STR, phenotypic lag for RIF), the manuscript does not provide a formal assessment of whether these additional parameters are warranted. As models become increasingly parameter-rich (incorporating allele-specific growth rates, allele-specific mutation probabilities, and phenotypic-delay parameters), the risk of overfitting rises. Model acceptance is based on achieving p > 0.1 when comparing simulated and experimental distributions, but no statistical model-selection framework (e.g., AIC/BIC comparison, likelihood-ratio tests, or cross-validation) is applied to show that more complex models are justified over simpler alternatives. Without such analyses, it is unclear whether the improved fit reflects genuine mechanistic insight or simply the increased flexibility afforded by additional parameters. Explicitly evaluating model parsimony would strengthen the conclusions and clarify whether each added mechanism is truly necessary to explain the data.

We provide p-values for each model fitting each type of data. Model selection is based on achieving all p-values above the (arbitrary) threshold of $p_{\mathrm{value}}=0.1$. We think that the more complex model selection framework mentioned by the Reviewer would essentially achieve the same (i.e., showed which model fits the data better) with little added value, as it usually very clear when one of our models does not fit the data ($p_{\mathrm{value}}$<0.001).

We agree with the Reviewer that the reason the most complex model (phenotypic lag) fits better is in part because it has two additional parameters to fit ($\omega$ and $q$). However, the main point we are trying to make here is (as discussed in response to Reviewer #1’s comment) not to explain the exact mechanism of resistance evolution but to show where and why a simple model fails in the case of each antibiotic, and find a minimal model that is biologically plausible and able to reproduce our experimental data.

See our response to Reviewer #1 for a more detailed discussion of this point, and the proposed change to the manuscript.

3) While the inclusion of CIP-induced filamentation and RIF-related phenotypic delay is biologically motivated, the functional forms used to represent these mechanisms (e.g., the time-dependent OD–cell density conversion factor; the intermediate “partially resistant” state) appear to be introduced post hoc to capture discrepancies between the initial model and the experimental data. The manuscript does not clearly justify why these particular formulations follow from independent biological theory or measurements, nor does it evaluate whether alternative mechanistic representations would fit the data equally well. As a result, it remains unclear to what extent these components provide mechanistic insight versus additional flexibility to improve the model fit. Strengthening the biological justification for these mathematical choices, or testing competing formulations, would make the interpretations more robust.

We stress (cf. the response to Reviewer #1) that our aim is to show what level of accuracy one may expect when comparing simple models with experiments, and how small- but significant modifications improve the accuracy of the models. It is not our intention to create mechanistic models, however all additions we propose are based on mechanisms already discussed in the literature, and we provide references to these observations. For example, phenotypic lag hypothesis dates back to 1940’s:

<http://www.jstor.org/stable/2984083>

PMID: 17247291

<https://doi.org/10.1073/pnas.35.1.1>,

with more recent work in Refs. [41,42]. Multiple alleles in RIF and CIP resistance have been observed in laboratory and in clinical isolates [33,34,47,48], see also

<https://link.springer.com/article/10.1186/s12866-023-02940-8>

<https://pmc.ncbi.nlm.nih.gov/articles/PMC10659180/>

<https://www.sciencedirect.com/science/article/abs/pii/S0924857903000360?via%3Dihub> . Filamentation is also a known phenomenon in response to fluoroquinolone antibiotics [36,37].

We believe that, in the field of modelling antibiotic resistance evolution, too much is relied on simple models that make only qualitative predictions. Here we are trying to elevate this comparison to a new level by making the comparison quantitative, and demonstrate that, while the simple model fails in many aspects, it can be easily amended to match the data. We think that it is these modified models, and not the bare birth-death-mutation model, that should be now considered “null models” in the field.

We have added a comment along these lines to Discussion.

Minor Concerns

1) Line 65: Reference 24 pertains to carcinogenesis theory. Having gone through it briefly, it is unclear how this relates to antibiotic resistance evolution. A brief justification or a more relevant citation would be helpful.

Reference [24] is a classical paper on birth-death processes, which is why we included it there. The reviewer is correct it does not discuss antibiotic resistance; we decided to delete it as the Lea and Coulson [23] paper is sufficient to introduce the model.

2) Line 89: While the model captures growth dynamics well, it is unclear whether evolutionary dynamics over many generations are sensitive to the parameters emphasized (this is also part of my major concern). Clarification would strengthen the argument.

We believe we have provided an answer to this question when addressing the major concern. We should also emphasize that our paper is only discussing the initial survival to the exposure of an antibiotic over only a few tens of generations.

3) Lines 113–118 (and other mentions of FLAS): The use of differently marked isogenic strains is interesting, but it is unclear how inferred FLAS values (e.g., 0 vs. 0.5) influence interpretation. What conceptual or biological difference should the reader take away?

A sentence in the paragraph mentioned by the Reviewer (“The use of a mixture of two easy-to-distinguish strains helped to determine the clonality of the final population.”) explains why we use this measure. Lines 208-212 (line numbers referring to the new manuscript) explain how to interpret FLAS.

There is no difference between the strains other than the fluorescence, see the modified sentence in line 130.

4) Line 135: Ensure consistency of units throughout the manuscript.

Different units (mg/ml or ng/ml) have been selected to avoid very different numeric values of antibiotic concentrations, for antibiotics for which the MIC differs by three orders of magnitude (CIP vs RIF and STR).

5) Line 217: Is a threshold of p < 0.1 considered conservative? A rationale would be helpful.

It is conservative in the sense that a model is rejected even if it slightly underfits the data. This helps to distinguish between models that just barely reproduce the data (p small but >>0.01) and models that are excellent fits (p on the order of one). This is already explained in lines 231-234, which we have now rephrased slightly.

6) Line 257: The phrase “…genetic to phenotypic resistance” is unclear; clarification is needed.

What we mean is that the simple models quantitatively reproduce growth, but they fail to reproduce the process of mutation and/or the emergence of phenotypic resistance from genetic mutations.

7) Line 279: The discussion of mutation rate changes under steady exponential growth versus transitions to stationary phase needs further explanation. How would growth-phase dynamics alter mutation rates?

We discuss in lines 503-508 why this could be, proposing that this is not necessary the result of an altered mutation rate but a result of phenotypic delay which affects the perceived (not the real) mutation rate. The real mutation rate also changes depending on the phase of growth; see for example DOI:10.1126/science.1087911 which claims the mutation rate increases 10x in the stationary phase. However, this would have had an opposite effect to what we observe (fewer resistant cells from experiments going into the stationary phase than from exponential growth experiments).

8) Lines 340–347: In Fig. 4, the mutation rate for mutS is 3.2 × 10^-9, while in the text it is mentioned as 4 × 10^-9. Which of these was used to fit the models? Also, the latter comes from small experiments, counting only normal-sized colonies. Selective counting may be subjective, and it is unclear what ‘normal’ means here; a more quantitative approach or discussion would improve confidence in these comparisons.

We apologize – the correct value is 3.2 x 10^-9 as in the figure, we have now corrected this in the text.

Regarding the difference between large and small colonies, the difference is quite striking and binary rather than a continuous spectrum of sizes. The small colonies are just a fraction of the size of the normal ones, and can easily be distinguished by eye.

9) Line 397: The term “semi-resistant” is somewhat confusing. Clarify whether the resistance mutation is recessive or dominant.

What we mean by “semi-resistant” is a phenotype that grows slower than a fully resistant one, but faster than the sensitive phenotype. We have clarified this in the manuscript (lines 412-414).

10) Lines 449–451: The discussion of “STR-dependent rpsL mutation” is confusing. Are the authors referring to streptomycin-dependent mutants i.e. mutants that need streptomycin for growth? Is there evidence that such mutants arose in these experiments?

Yes, we mean that the mutants require streptomycin for growth; such mutants have been described in the literature [32,46]. However, since we have not investigated our specific mutants in great detail, we have decided to tone down the message and changed the line to “thus rpsL mutations which are STR-dependant are more likely to be selected for”.

11) Figure 4C: The headers for rows 2 and 3 in the RIF panel appear identical; please verify.

We apologize for this. We have removed “lag” from the 2nd column.

12) Line 536: Given that only 25% of the population is retained at each dilution step, one might expect the effective mutation supply in bioreactor populations to be lower than in fluctuation assays. Do the authors’ estimates account for this?

It is actually the other way around: 75% of the population is retained in each dilution step, in agreement with what the sentence in lines 607-608 says: “Each dilution replaces 25% of the culture volume with fresh medium”. Our model accounts for this because it explicitly models dilution exactly as it happens in the bioreactor.

13) Lines 562–563: The sentence is difficult to follow. Were cultures stopped at different time points to ensure that all reached OD 0.1? Clarification is needed.

We have changed the sentence to explain it better why different cultures had to be stopped at slightly different times.

14) Line 655: The equation needs more explanation; e.g., what do a and af represent?

$a,b,c$ are the free parameters of the fit and $f$ is the dilution factor. We have added a brief explanation below the formula.

15) Line 710: It is unclear what represents here and how its values were determined.

We have added an additional explanation.
