# Supplementary material for: Simple birth-death-mutation models predict some—but not all—aspects of the experimental evolution of antibiotic resistance
Source: PLoS Comput Biol. 2026 Aug 28;22(8):e1014666. doi: 10.1371/journal.pcbi.1014666 (PMC13552961; doi:10.1371/journal.pcbi.1014666)
Supplement: Supplementary file 9 [file pcbi.1014666.s012.docx]

We thank the Reviewers for their comments. Please find below a response to each comment. Reviewer’s original comments are in black, our response is in blue.

Note that we have moved Section “Broader implications” from “Discussion” to the end of the “Results” section. This has affected line numbers. Our references to specific lines in the replies apply to the new, clean manuscript (without tracked changes).

**Reviewer #1:** I consider the authors have addressed my comments appropriately. I still think, however, that my first point regarding making more explicit what is novel and the implications of their findings needs more work. Their new section on “Broader implications” does not quite address this matter.

One final comment: the sentence “This is perhaps not entirely unexpected, but we have never seen it articulated this clearly” in lines 513-514 strikes me as somewhat overstated and could be rephrased more cautiously.

I leave these comments as optional suggestions for the authors to consider. All in all, I think the manuscript is in good condition and suitable for publication.

We have changed this sentence (lines 568-570) to “This is perhaps not entirely unexpected, but we think this point is worth articulating to ensure that this simple model is used with caution when applied to real data.”

**Reviewer #2:** I have no further comments.

We thank the Reviewer for their constructive comments during the previous revision.

**Reviewer #3:** I appreciate the authors' clarifications regarding the rationale behind the progressive refinement of their models. In particular, many quantities incorporated into the more complex formulations (e.g., allele-specific growth rates and mutation probabilities) were determined independently rather than fitted to the same data used for model evaluation, suggesting that the risk of classical overfitting is likely limited. Likewise, the additional components introduced into the models (filamentation, phenotypic lag, multiple resistant alleles) are grounded in established biological observations and are intended as minimal extensions required to restore quantitative agreement between model predictions and experimental data, rather than as mechanistic discoveries in themselves.

My remaining concerns relate less to the validity of these biological mechanisms and more to the interpretation, selection, and generalizability of the resulting models. I have listed these concerns below.

1) The criterion used to select among models, i.e., requiring all comparisons between simulated and experimental observables to achieve p-values greater than 0.1, is ultimately a heuristic rather than a formal model-selection framework. While the differences between models are often substantial (for example, when simpler models clearly fail with p-values < 0.001), and therefore more formal approaches such as information-theoretic model comparison might well arrive at the same conclusions, the current approach does not explicitly address model parsimony or provide a principled basis for choosing between alternative models that may all adequately reproduce the data. A brief discussion of this limitation would improve transparency and help readers understand how the authors define the "minimal" model required to explain each antibiotic-specific dataset.

We reiterate that it is not our intention to perform a formal model selection, but to show where and why the simple B-D-M model fails in the case of each antibiotic, and show an example minimal model that is biologically plausible and able to reproduce our experimental data. The approach we have chosen achieves that and is therefore sufficient for our purpose, even though it is not as formal as other approaches.

We would also like to point out that any statistical model comparison ultimately includes a criterion which cannot be derived mathematically from first principles. This is why statistics is both a science and an art, and why there is no single universally accepted way of model rejection and model comparison, and different researchers use different approaches (p-values, Akaike Information Criterion, Bayesian Information Criterion etc). Being pragmatic, we believe that the best approach is to select a tool sufficient for the question asked even though there may be better (more formal, more accurate etc.) tools available.

Since we believe our intentions have been made very clear already in the previous revision, we have now added only a short comment in the Discussion, lines 573-575, to address the problem of model selection.

2) Although the added mechanisms are biologically motivated, the specific mathematical implementations chosen to represent them are not necessarily unique. I do not expect an exhaustive exploration of alternative formulations. Although the authors stress that their primary goal is not to develop mechanistic models, the manuscript would benefit from acknowledging that different, yet biologically plausible, representations of the same process (e.g., phenotypic lag or filamentation) might lead to quantitatively different predictions. Clarifying the extent to which the principal conclusions depend on these particular implementations would strengthen confidence that the observed improvements reflect general mechanistic effects rather than sensitivity to specific modelling choices.

We have commented on this in the Discussion, lines 575-581.

3) I would encourage the authors to temper some of the broader translational and general statements made throughout the manuscript. For example, statements such as "Our work sets a baseline for predictive models and clarifies the scale of deviations that can be expected when extensions beyond the birth–death–mutation model are ignored" or "when considering the outcome of treating a bacterial population with an antibiotic, it is important to take into account the initial response of the population, the fitness of different resistant alleles, and phenotypic delay" appear stronger than what is directly supported by the present study.

We have changed these to (lines 570-571)

"Our work clarifies the scale of deviations that can be expected when extensions beyond the birth–death–mutation model are ignored."

and (lines 588-591)

"… when modelling antibiotic treatment, the initial response of the population, the fitness of different resistant alleles, and phenotypic delay should be considered as important factors that may significantly affect the model prediction."

One of the most interesting findings of this work is that the same simple birth–death–mutation framework fails for different antibiotics for different reasons. Restoring quantitative agreement required introducing distinct antibiotic-specific mechanisms, and identifying these mechanisms relied on detailed experimental characterization of each system. Consequently, the study convincingly demonstrates that simple models may not be quantitatively predictive and that incorporating biologically relevant processes can substantially improve model performance. However, it is less clear that the particular mechanisms identified here can be generalized as a baseline set of factors that should routinely be incorporated into predictive models of resistance evolution or personalized antibiotic therapy.

Indeed, the results may equally support a more cautious interpretation: achieving predictive accuracy may require prior knowledge of which biological processes are important for a given antibiotic–pathogen system, and these processes may differ substantially across contexts. I do not view this as a weakness of the study; rather, I think it is one of its most important insights.

We thank the Reviewer for this suggestion. This is what we have been trying to say but perhaps not clearly enough. We have added the sentence (lines 586-588).

“Indeed, accurate predictions for all models of antibiotic resistance may depend on prior knowledge of the biological processes governing a particular antibiotic–pathogen system, which can differ substantially between contexts.”

Explicitly acknowledging this limitation and tempering broader claims of general applicability would help align the conclusions with the scope of the evidence presented.

We hope the above changes are sufficient in that respect.

4) Finally, I found the newly added discussion of broader implications and Figure 5 valuable, particularly because it illustrates that these mechanisms can, in principle, influence resistance outcomes. However, these simulations are based on a single illustrative parameter set and are explicitly not intended as a comprehensive exploration of parameter space. At the same time, the discussion (Lines 544–546) describes how these illustrative scenarios might mimic clinical infections. This makes it difficult to assess how broadly these conclusions can be applied. A systematic sensitivity analysis across biologically realistic parameter ranges would substantially strengthen these claims by identifying which qualitative behaviours are robust. I do not consider such analyses essential for publication. However, if they are beyond the scope of the present study, I encourage the authors to more explicitly acknowledge the limitations of these illustrative simulations and clarify that the clinical and experimental implications should be interpreted as hypotheses generated by the model, rather than general predictions established by it.

We have added (lines 485-487) that “Moreover, these illustrative examples should be interpreted as hypotheses generated by the model, rather than general predictions applicable to all models”.

Overall, I do not view any of these points as barriers to publication. Rather, I believe that explicitly discussing these limitations would help readers better understand the strengths, scope, and intended use of the proposed modelling framework.
