# Supplementary material for: Simple birth-death-mutation models predict some—but not all—aspects of the experimental evolution of antibiotic resistance
Source: PLoS Comput Biol. 2026 Aug 28;22(8):e1014666. doi: 10.1371/journal.pcbi.1014666 (PMC13552961; doi:10.1371/journal.pcbi.1014666)
Supplement: Peer review history

# Peer review: Decision Letter 0

**DOI:** 10.1371/journal.pcbi.1014666.r001

**Authors:** Natalia Komarova, Ville Mustonen

28 Nov 2025

Simple theoretical models predict some - but not all - aspects of the experimental evolution of antibiotic resistance

PLOS Computational Biology

Dear Dr. Waclaw,

Thank you for submitting your manuscript to PLOS Computational Biology. After careful consideration, we feel that it has merit but does not fully meet PLOS Computational Biology's publication criteria as it currently stands. Therefore, we invite you to submit a revised version of the manuscript that addresses the points raised during the review process.

Please submit your revised manuscript by Jan 28 2026 11:59PM. If you will need more time than this to complete your revisions, please reply to this message or contact the journal office at ploscompbiol@plos.org. When you're ready to submit your revision, log on to https://www.editorialmanager.com/pcompbiol/ and select the 'Submissions Needing Revision' folder to locate your manuscript file.

Please include the following items when submitting your revised manuscript:

* A rebuttal letter that responds to each point raised by the editor and reviewer(s). You should upload this letter as a separate file labeled 'Response to Reviewers'. This file does not need to include responses to formatting updates and technical items listed in the 'Journal Requirements' section below.

* A marked-up copy of your manuscript that highlights changes made to the original version. You should upload this as a separate file labeled 'Revised Manuscript with Track Changes'.

* An unmarked version of your revised paper without tracked changes. You should upload this as a separate file labeled 'Manuscript'.

If you would like to make changes to your financial disclosure, competing interests statement, or data availability statement, please make these updates within the submission form at the time of resubmission. Guidelines for resubmitting your figure files are available below the reviewer comments at the end of this letter

We look forward to receiving your revised manuscript.

Kind regards,

Ville Mustonen

Academic Editor

PLOS Computational Biology

Natalia Komarova

Section Editor

PLOS Computational Biology

**Additional Editor Comments:**

Thank you for submitting your manuscript to PLOS CB. All three reviewers see clear merit in your work as do I. Please take their comments and concerns into consideration while revising to further clarify and improve the manuscript.

**Journal Requirements:**

1) Please ensure that the CRediT author contributions listed for every co-author are completed accurately and in full.

At this stage, the following Authors/Authors require contributions: Elin Lilja, Rosalind Allen, and Bartlomiej Waclaw. Please ensure that the full contributions of each author are acknowledged in the "Add/Edit/Remove Authors" section of our submission form.

The list of CRediT author contributions may be found here: https://journals.plos.org/ploscompbiol/s/authorship#loc-author-contributions

2) Please provide an Author Summary. This should appear in your manuscript between the Abstract (if applicable) and the Introduction, and should be 150-200 words long. The aim should be to make your findings accessible to a wide audience that includes both scientists and non-scientists. Sample summaries can be found on our website under Submission Guidelines:

https://journals.plos.org/ploscompbiol/s/submission-guidelines#loc-parts-of-a-submission

3) Please upload all main figures as separate Figure files in .tif or .eps format. For more information about how to convert and format your figure files please see our guidelines:

https://journals.plos.org/ploscompbiol/s/figures

4) We have noticed that you have uploaded Supporting Information files, but you have not included a list of legends. Please add a full list of legends for your Supporting Information files after the references list.

5) Please amend your detailed Financial Disclosure statement. This is published with the article. It must therefore be completed in full sentences and contain the exact wording you wish to be published.

- State the initials, alongside each funding source, of each author to receive each grant. For example: "This work was supported by the National Institutes of Health (####### to AM; ###### to CJ) and the National Science Foundation (###### to AM)."

- State what role the funders took in the study. If the funders had no role in your study, please state: "The funders had no role in study design, data collection and analysis, decision to publish, or preparation of the manuscript.".

If you did not receive any funding for this study, please simply state: u201cThe authors received no specific funding for this work.u201d

**Reviewers' comments:**

Reviewer's Responses to Questions

**Comments to the Authors:**

**Please note here if the review is uploaded as an attachment.**

Reviewer #1: The manuscript presents clear analyses and reports interesting results. I appreciate the transparent exposition and the careful presentation of simulations and comparisons. I appreciate the manuscript’s coupling of experiments and theory.

Major comments:

- While the findings are interesting, it remains unclear how broadly they generalize or how they should be used in practice. The take-home message that birth-death-mutation (BDM) models can predict some aspects of resistance evolution but not others, is somewhat unsurprising. It would help if the authors articulated what is novel and useful here, and concluded with concrete guidance on use: for example, should BDM models be treated as null models for baseline predictions, stress-tests/sanity checks, or quick screening tools before deploying richer frameworks?

- The introduction needs more context. There are several other models that have been employed to study resistance evolution, for example, branching processes, models involving division times between cells, diffusion models, consumer-resource models, etc. It may also help to note common modeling extensions that are relevant in practice: PK/PD coupling, non-genetic versus genetic resistance, and drug-specific mechanisms. Also, it would be useful to list the assumptions behind BDM models (e.g., well-mixed population, no epistasis, no competition, absence of spatial structure, etc.).

- What is the reason for using the tau-leaping algorithm in your study? My guess is that with exact algorithms (such as the Gillespie algorithm), the simulations are very costly at the population sizes considered. While the tau-leaping algorithm is a useful tool that speeds up simulations, its approximations can introduce bias. Part of the discrepancies between theory and data that you observe could come from using this algorithm. Have the authors validated this algorithm for the cases studied? Including a small-scale benchmark against the Gillespie algorithm (on subsets where exact simulation is feasible) would strengthen confidence in your results.

- A weak point of this study is that modifications to the model are introduced only after observing discrepancies. The modifications are justified to some extent, but several different modifications could have been considered to produce similar observables. Adding them post-hoc can fit data but weakens generality. I understand the main purpose of this paper is not to explain the mechanisms behind, but the paper could benefit from discussing these points. Besides, adding extra modifications has the risk of overfitting data. Perhaps the author could comment on that as well. If possible, clarifying which modifications are necessary versus merely sufficient would help readers gauge portability to new datasets.

Minor comments:

- The paper is generally very clear, but Figure 4 is visually crowded. Consider increasing the gap between panels and increasing the font size. I recommend also adding an x-axis to the second-row panels.

- Consider improving the notation in your equations. Perhaps a piecewise notation with intervals could improve their readability.

Reviewer #2: Summary:

In this paper, the authors investigate evolution of resistance to three antibiotics in bioreactor cultures that allow for continuous exponential growth. The authors attempt to rigorously test how well a type of birth-death-mutation process can quantitatively explain the observed experimental results by looking at some more nuanced observables such as regrowth time distributions. While numerical simulations of the mathematical model largely match with the observed trajectories, the authors show that some quantitative discrepancies arise depending on the used antibiotic and then present some extensions to the model that can better explain these deviations.

By reading the paper it is apparent that this work is of high quality. Although there is limited new computational or mathematical advances, the experimental setup is quite impressive, and I agree with the authors that biology needs more rigorous application of quantitative methods. While I am not sure I fully agree with all the reached conclusions and the overall framing of the paper (please see more detailed comments below), I think it is in principle acceptable to PLOS Computational Biology without needing any major changes.

Comments:

1. The codes cannot be accessed from the provided link. Note that this journal requires all the materials to be made available for review.

2. The paper is written in a way that presents the birth-death-mutation process as an agreed “null model” that the authors then aim to reject. While this is certainly a refreshing take in this context, I am not convinced that this is the case. Firstly, because there are many different flavours and more subtle versions of such models, but also other simple models that could be used that have been developed especially in the evolutionary rescue literature. Thus, I am not sure what the quite small discrepancies of this particular model ultimately tell us. Rigorous hypothesis testing first needs a clear null model or proposed hypothesis/mechanism, which I don’t think is very clear here. What is the precise assumption of the model that we can now reject?

3. Continuing on this, my reading of the paper is that simple models, indeed simple birth-death-mutation models, can explain the data. The main difficulties are more related to the challenge of translating and inferring the model variables and parameters from the biological observables. Certainly, the authors haven’t shown that an even simpler model could not explain everything without needing too many modifications for each antibiotic. I think the main title is in this sense a bit misleading and would prefer the short title instead (“Testing simple models of antibiotic resistance evolution”).

4. I think the observation that the inferred mutation rate from the fluctuation test is different from the apparent mutation rate in the bioreactor is one of the more interesting findings of the paper that could be expanded as this is likely important both biologically as well as in terms of thinking about the experiments. This could also be nicely linked to theory.

5. Why is the number of replicates for each antibiotic different? Was some data excluded from the analysis?

6. How are the probability distributions for the regrowth times predicted by the model computed exactly in Fig. 2D? Their shape looks a bit weird to me, and given the simplicity of the model, some analytical results might also be possible.

7. In Fig. 2E, what is the meaning of the error bars? Doesn’t one simply compute one number for the empirical probability of regrowth from all the replicates?

Reviewer #3: The study by Lilja et al. investigates how mathematical models describing experimental evolution of antibiotic resistance can be made more accurate. The authors examine resistance evolution in a controlled bioreactor system, fitting model predictions to their empirical results. Overall, the manuscript is well-written, and the experiments appear technically sound. However, I have several major and minor concerns that should be addressed before publication.

Major Concerns

1) The manuscript demonstrates that including mechanistic components improves the quantitative match between model predictions and experimental measurements. However, it remains unclear what these refinements teach us about the evolution of resistance itself. Specifically, it is not shown whether these parameters, once included, actually alter the evolutionary outcome or trajectory in a meaningful way. As written, the narrative primarily demonstrates that including mechanistic realism improves predictive accuracy, which is interesting but an intuitive result. What is missing is a demonstration that these mechanisms affect the evolutionary dynamics, rather than merely helping the model fit the data post hoc. For example, does varying the phenotypic-lag parameter change the probability of resistance emergence, the distribution of resistant alleles, or the timing of regrowth in a way that yields new insight into RIF resistance evolution? Will it change evolutionary outcomes in experimental evolution studies using these antibiotics? Without such analyses, it is difficult to see how these additions reveal new principles about antibiotic action or resistance evolution. I encourage the authors to show how inferred parameters shape long-term evolutionary outcomes, or to explicitly discuss the conceptual advances arising from these refinements.

2) While I appreciate the authors’ stepwise refinement of the mathematical models by incorporating mechanistically motivated features (e.g., filamentation for CIP, delayed initial response for STR, phenotypic lag for RIF), the manuscript does not provide a formal assessment of whether these additional parameters are warranted. As models become increasingly parameter-rich (incorporating allele-specific growth rates, allele-specific mutation probabilities, and phenotypic-delay parameters), the risk of overfitting rises. Model acceptance is based on achieving p > 0.1 when comparing simulated and experimental distributions, but no statistical model-selection framework (e.g., AIC/BIC comparison, likelihood-ratio tests, or cross-validation) is applied to show that more complex models are justified over simpler alternatives. Without such analyses, it is unclear whether the improved fit reflects genuine mechanistic insight or simply the increased flexibility afforded by additional parameters. Explicitly evaluating model parsimony would strengthen the conclusions and clarify whether each added mechanism is truly necessary to explain the data.

3) While the inclusion of CIP-induced filamentation and RIF-related phenotypic delay is biologically motivated, the functional forms used to represent these mechanisms (e.g., the time-dependent OD–cell density conversion factor; the intermediate “partially resistant” state) appear to be introduced post hoc to capture discrepancies between the initial model and the experimental data. The manuscript does not clearly justify why these particular formulations follow from independent biological theory or measurements, nor does it evaluate whether alternative mechanistic representations would fit the data equally well. As a result, it remains unclear to what extent these components provide mechanistic insight versus additional flexibility to improve the model fit. Strengthening the biological justification for these mathematical choices, or testing competing formulations, would make the interpretations more robust.

Minor Concerns

1) Line 65: Reference 24 pertains to carcinogenesis theory. Having gone through it briefly, it is unclear how this relates to antibiotic resistance evolution. A brief justification or a more relevant citation would be helpful.

2) Line 89: While the model captures growth dynamics well, it is unclear whether evolutionary dynamics over many generations are sensitive to the parameters emphasized (this is also part of my major concern). Clarification would strengthen the argument.

3) Lines 113–118 (and other mentions of FLAS): The use of differently marked isogenic strains is interesting, but it is unclear how inferred FLAS values (e.g., 0 vs. 0.5) influence interpretation. What conceptual or biological difference should the reader take away?

4) Line 135: Ensure consistency of units throughout the manuscript.

5) Line 217: Is a threshold of p < 0.1 considered conservative? A rationale would be helpful.

6) Line 257: The phrase “…genetic to phenotypic resistance” is unclear; clarification is needed.

7) Line 279: The discussion of mutation rate changes under steady exponential growth versus transitions to stationary phase needs further explanation. How would growth-phase dynamics alter mutation rates?

8) Lines 340–347: In Fig. 4, the mutation rate for mutS is 3.2 × 10^-9, while in the text it is mentioned as 4 × 10^-9. Which of these was used to fit the models? Also, the latter comes from small experiments, counting only normal-sized colonies. Selective counting may be subjective, and it is unclear what ‘normal’ means here; a more quantitative approach or discussion would improve confidence in these comparisons.

9) Line 397: The term “semi-resistant” is somewhat confusing. Clarify whether the resistance mutation is recessive or dominant.

10) Lines 449–451: The discussion of “STR-dependent rpsL mutation” is confusing. Are the authors referring to streptomycin-dependent mutants i.e. mutants that need streptomycin for growth? Is there evidence that such mutants arose in these experiments?

11) Figure 4C: The headers for rows 2 and 3 in the RIF panel appear identical; please verify.

12) Line 536: Given that only 25% of the population is retained at each dilution step, one might expect the effective mutation supply in bioreactor populations to be lower than in fluctuation assays. Do the authors’ estimates account for this?

13) Lines 562–563: The sentence is difficult to follow. Were cultures stopped at different time points to ensure that all reached OD 0.1? Clarification is needed.

14) Line 655: The equation needs more explanation; e.g., what do a and af represent?

15) Line 710: It is unclear what represents here and how its values were determined.

**********

**Have the authors made all data and (if applicable) computational code underlying the findings in their manuscript fully available?**

The PLOS Data policy requires authors to make all data and code underlying the findings described in their manuscript fully available without restriction, with rare exception (please refer to the Data Availability Statement in the manuscript PDF file). The data and code should be provided as part of the manuscript or its supporting information, or deposited to a public repository. For example, in addition to summary statistics, the data points behind means, medians and variance measures should be available. If there are restrictions on publicly sharing data or code —e.g. participant privacy or use of data from a third party—those must be specified.

Reviewer #1: Yes

Reviewer #2: **No:** code not accessible

Reviewer #3: Yes

**********

PLOS authors have the option to publish the peer review history of their article (what does this mean?). If published, this will include your full peer review and any attached files.

If you choose “no”, your identity will remain anonymous but your review may still be made public.

**Do you want your identity to be public for this peer review?**  For information about this choice, including consent withdrawal, please see our Privacy Policy.

Reviewer #1: No

Reviewer #2: No

Reviewer #3: No

[NOTE: If reviewer comments were submitted as an attachment file, they will be attached to this email and accessible via the submission site. Please log into your account, locate the manuscript record, and check for the action link "View Attachments". If this link does not appear, there are no attachment files.]

**Figure resubmission:**

**Reproducibility:**

?>

# Author comment: Author response to Decision Letter 1

**DOI:** 10.1371/journal.pcbi.1014666.r002

19 May 2026

Attachment Submitted filename: reply_2.docx

# Peer review: Decision Letter 1

**DOI:** 10.1371/journal.pcbi.1014666.r003

**Authors:** Natalia Komarova, Ville Mustonen

18 Jun 2026

PCOMPBIOL-D-25-02099R1

Simple birth-death-mutation models predict some - but not all - aspects of the experimental evolution of antibiotic resistance

PLOS Computational Biology

Dear Dr. Waclaw,

Thank you for submitting your manuscript to PLOS Computational Biology. After careful consideration, we feel that it has merit but does not fully meet PLOS Computational Biology's publication criteria as it currently stands. Therefore, we invite you to submit a revised version of the manuscript that addresses the points raised during the review process.

Please submit your revised manuscript by Aug 18 2026 11:59PM. If you will need more time than this to complete your revisions, please reply to this message or contact the journal office at ploscompbiol@plos.org. When you're ready to submit your revision, log on to https://www.editorialmanager.com/pcompbiol/ and select the 'Submissions Needing Revision' folder to locate your manuscript file.

Please include the following items when submitting your revised manuscript:

* A letter that responds to each point raised by the editor and reviewer(s). You should upload this letter as a separate file labeled 'Response to Reviewers'. This file does not need to include responses to formatting updates and technical items listed in the 'Journal Requirements' section below.

* A marked-up copy of your manuscript that highlights changes made to the original version. You should upload this as a separate file labeled 'Revised Manuscript with Track Changes'.

* An unmarked version of your revised paper without tracked changes. You should upload this as a separate file labeled 'Manuscript'.

If you would like to make changes to your financial disclosure, competing interests statement, or data availability statement, please make these updates within the submission form at the time of resubmission. Guidelines for resubmitting your figure files are available below the reviewer comments at the end of this letter.

As the corresponding author, your ORCID iD is verified in the submission system and will appear in the published article. PLOS supports the use of ORCID, and we encourage all coauthors to register for an ORCID iD and use it as well. Please encourage your coauthors to verify their ORCID iD within the submission system before final acceptance, as unverified ORCID iDs will not appear in the published article. Only the individual author can complete the verification step; PLOS staff cannot verify ORCID iDs on behalf of authors.

We look forward to receiving your revised manuscript.

Kind regards,

Ville Mustonen

Academic Editor

PLOS Computational Biology

Natalia Komarova

Section Editor

PLOS Computational Biology

**Additional Editor Comments (if provided):**

Dear Authors,

Thank you for your revision. One reviewer is fully satisfied, the two other reviewers do not have any major open issues but have made valuable points that I would like you to address to improve the work further. Please try to complete these swiftly, I do not expect to send your new revision again to the reviewers so that the manuscript can then proceed to publication stages rapidly.

**Reviewers' comments:**

Reviewer's Responses to Questions

**Comments to the Authors:**

**Please note here if the review is uploaded as an attachment.**

Reviewer #1: I consider the authors have addressed my comments appropriately. I still think, however, that my first point regarding making more explicit what is novel and the implications of their findings needs more work. Their new section on “Broader implications” does not quite address this matter.

One final comment: the sentence “This is perhaps not entirely unexpected, but we have never seen it articulated this clearly” in lines 513-514 strikes me as somewhat overstated and could be rephrased more cautiously.

I leave these comments as optional suggestions for the authors to consider. All in all, I think the manuscript is in good condition and suitable for publication.

Reviewer #2: I have no further comments.

Reviewer #3: I appreciate the authors' clarifications regarding the rationale behind the progressive refinement of their models. In particular, many quantities incorporated into the more complex formulations (e.g., allele-specific growth rates and mutation probabilities) were determined independently rather than fitted to the same data used for model evaluation, suggesting that the risk of classical overfitting is likely limited. Likewise, the additional components introduced into the models (filamentation, phenotypic lag, multiple resistant alleles) are grounded in established biological observations and are intended as minimal extensions required to restore quantitative agreement between model predictions and experimental data, rather than as mechanistic discoveries in themselves.

My remaining concerns relate less to the validity of these biological mechanisms and more to the interpretation, selection, and generalizability of the resulting models. I have listed these concerns below.

1) The criterion used to select among models, i.e., requiring all comparisons between simulated and experimental observables to achieve p-values greater than 0.1, is ultimately a heuristic rather than a formal model-selection framework. While the differences between models are often substantial (for example, when simpler models clearly fail with p-values < 0.001), and therefore more formal approaches such as information-theoretic model comparison might well arrive at the same conclusions, the current approach does not explicitly address model parsimony or provide a principled basis for choosing between alternative models that may all adequately reproduce the data. A brief discussion of this limitation would improve transparency and help readers understand how the authors define the "minimal" model required to explain each antibiotic-specific dataset.

2) Although the added mechanisms are biologically motivated, the specific mathematical implementations chosen to represent them are not necessarily unique. I do not expect an exhaustive exploration of alternative formulations. Although the authors stress that their primary goal is not to develop mechanistic models, the manuscript would benefit from acknowledging that different, yet biologically plausible, representations of the same process (e.g., phenotypic lag or filamentation) might lead to quantitatively different predictions. Clarifying the extent to which the principal conclusions depend on these particular implementations would strengthen confidence that the observed improvements reflect general mechanistic effects rather than sensitivity to specific modelling choices.

3) I would encourage the authors to temper some of the broader translational and general statements made throughout the manuscript. For example, statements such as "Our work sets a baseline for predictive models and clarifies the scale of deviations that can be expected when extensions beyond the birth–death–mutation model are ignored" or "when considering the outcome of treating a bacterial population with an antibiotic, it is important to take into account the initial response of the population, the fitness of different resistant alleles, and phenotypic delay" appear stronger than what is directly supported by the present study.

One of the most interesting findings of this work is that the same simple birth–death–mutation framework fails for different antibiotics for different reasons. Restoring quantitative agreement required introducing distinct antibiotic-specific mechanisms, and identifying these mechanisms relied on detailed experimental characterization of each system. Consequently, the study convincingly demonstrates that simple models may not be quantitatively predictive and that incorporating biologically relevant processes can substantially improve model performance. However, it is less clear that the particular mechanisms identified here can be generalized as a baseline set of factors that should routinely be incorporated into predictive models of resistance evolution or personalized antibiotic therapy.

Indeed, the results may equally support a more cautious interpretation: achieving predictive accuracy may require prior knowledge of which biological processes are important for a given antibiotic–pathogen system, and these processes may differ substantially across contexts. I do not view this as a weakness of the study; rather, I think it is one of its most important insights. Explicitly acknowledging this limitation and tempering broader claims of general applicability would help align the conclusions with the scope of the evidence presented.

4) Finally, I found the newly added discussion of broader implications and Figure 5 valuable, particularly because it illustrates that these mechanisms can, in principle, influence resistance outcomes. However, these simulations are based on a single illustrative parameter set and are explicitly not intended as a comprehensive exploration of parameter space. At the same time, the discussion (Lines 544–546) describes how these illustrative scenarios might mimic clinical infections. This makes it difficult to assess how broadly these conclusions can be applied. A systematic sensitivity analysis across biologically realistic parameter ranges would substantially strengthen these claims by identifying which qualitative behaviours are robust. I do not consider such analyses essential for publication. However, if they are beyond the scope of the present study, I encourage the authors to more explicitly acknowledge the limitations of these illustrative simulations and clarify that the clinical and experimental implications should be interpreted as hypotheses generated by the model, rather than general predictions established by it.

Overall, I do not view any of these points as barriers to publication. Rather, I believe that explicitly discussing these limitations would help readers better understand the strengths, scope, and intended use of the proposed modelling framework.

**********

**Have the authors made all data and (if applicable) computational code underlying the findings in their manuscript fully available?**

The PLOS Data policy requires authors to make all data and code underlying the findings described in their manuscript fully available without restriction, with rare exception (please refer to the Data Availability Statement in the manuscript PDF file). The data and code should be provided as part of the manuscript or its supporting information, or deposited to a public repository. For example, in addition to summary statistics, the data points behind means, medians and variance measures should be available. If there are restrictions on publicly sharing data or code —e.g. participant privacy or use of data from a third party—those must be specified.

Reviewer #1: Yes

Reviewer #2: None

Reviewer #3: Yes

**********

PLOS authors have the option to publish the peer review history of their article (what does this mean?). If published, this will include your full peer review and any attached files.

If you choose “no”, your identity will remain anonymous but your review may still be made public.

**Do you want your identity to be public for this peer review?**  For information about this choice, including consent withdrawal, please see our Privacy Policy.

Reviewer #1: No

Reviewer #2: No

Reviewer #3: No

[NOTE: If reviewer comments were submitted as an attachment file, they will be attached to this email and accessible via the submission site. Please log into your account, locate the manuscript record, and check for the action link "View Attachments". If this link does not appear, there are no attachment files.]

**Figure resubmission:**

**Reproducibility:**

To enhance the reproducibility of your results, we recommend that authors of applicable studies deposit laboratory protocols in protocols.io, where a protocol can be assigned its own identifier (DOI) such that it can be cited independently in the future. Additionally, PLOS ONE offers an option to publish peer-reviewed clinical study protocols. Read more information on sharing protocols at https://plos.org/protocols?utm_medium=editorial-email&utm_source=authorletters&utm_campaign=protocols

# Author comment: Author response to Decision Letter 2

**DOI:** 10.1371/journal.pcbi.1014666.r004

31 Jul 2026

Attachment Submitted filename: reply_final.docx

# Editor report: Decision Letter 2

**DOI:** 10.1371/journal.pcbi.1014666.r005

**Authors:** Natalia Komarova, Ville Mustonen

4 Aug 2026

Dear Dr Waclaw,

We are pleased to inform you that your manuscript 'Simple birth-death-mutation models predict some - but not all - aspects of the experimental evolution of antibiotic resistance' has been provisionally accepted for publication in PLOS Computational Biology.

Before your manuscript can be formally accepted you will need to complete some formatting changes, which you will receive in a follow up email. A member of our team will be in touch with a set of requests.

Please note that your manuscript will not be scheduled for publication until you have made the required changes, so a swift response is appreciated.

IMPORTANT: The editorial review process is now complete. PLOS will only permit corrections to spelling, formatting or significant scientific errors from this point onwards. Requests for major changes, or any which affect the scientific understanding of your work, will cause delays to the publication date of your manuscript.

Should you, your institution's press office or the journal office choose to press release your paper, you will automatically be opted out of early publication. We ask that you notify us now if you or your institution is planning to press release the article. All press must be co-ordinated with PLOS.

Thank you again for supporting Open Access publishing; we are looking forward to publishing your work in PLOS Computational Biology.

Best regards,

Ville Mustonen

Academic Editor

PLOS Computational Biology

Natalia Komarova

Section Editor

PLOS Computational Biology

***********************************************************

# Editor report: Acceptance letter

**DOI:** 10.1371/journal.pcbi.1014666.r006

**Authors:** Natalia Komarova, Ville Mustonen

PCOMPBIOL-D-25-02099R2

Simple birth-death-mutation models predict some - but not all - aspects of the experimental evolution of antibiotic resistance

Dear Dr Waclaw,

I am pleased to inform you that your manuscript has been formally accepted for publication in PLOS Computational Biology. Your manuscript is now with our production department and you will be notified of the publication date in due course.

The corresponding author will soon be receiving a typeset proof for review, to ensure errors have not been introduced during production. Please review the PDF proof of your manuscript carefully, as this is the last chance to correct any errors. Please note that major changes, or those which affect the scientific understanding of the work, will likely cause delays to the publication date of your manuscript.

Soon after your final files are uploaded, unless you have opted out, the early version of your manuscript will be published online. The date of the early version will be your article's publication date. The final article will be published to the same URL, and all versions of the paper will be accessible to readers.

For Research, Software, and Methods articles, you will receive an invoice from PLOS for your publication fee after your manuscript has reached the completed accept phase. If you receive an email requesting payment before acceptance or for any other service, this may be a phishing scheme. Learn how to identify phishing emails and protect your accounts at https://explore.plos.org/phishing.

Thank you again for supporting PLOS Computational Biology and open-access publishing. We are looking forward to publishing your work!

With kind regards,

Sharmila Kamatchi

PLOS Computational Biology | Carlyle House, Carlyle Road, Cambridge CB4 3DN | United Kingdom ploscompbiol@plos.org | Phone +44 (0) 1223-442824 | ploscompbiol.org | @PLOSCompBiol
